# An effective prognostic model for assessing prognosis of non-small cell lung cancer with brain metastases

**DOI:** 10.3389/fgene.2023.1156322

**Published:** 2023-04-13

**Authors:** Rong Wang, Xing Zhang, Changshou He, Wei Guo

**Affiliations:** ^1^ Respiratory department, Shanxi Cancer Hospital, Taiyuan, China; ^2^ Department of Oncology, HaploX Biotechnology, Shenzhen, China

**Keywords:** lung cancer, NSCLC, brain metastases, chemotherapy drugs, RiskScore model, prognosis

## Abstract

**Background:** Brain metastasis, with an incidence of more than 30%, is a common complication of non-small cell lung cancer (NSCLC). Therefore, there is an urgent need for an assessment method that can effectively predict brain metastases in NSCLC and help understand its mechanism.

**Materials and methods:** GSE30219, GSE31210, GSE37745, and GSE50081 datasets were downloaded from the GEO database and integrated into a dataset (GSE). The integrated dataset was divided into the training and test datasets. TCGA-NSCLC dataset was regarded as an independent verification dataset. Here, the limma R package was used to identify the differentially expression genes (DEGs). Importantly, the RiskScore model was constructed using univariate Cox regression analysis and least absolute shrinkage and selection operator (LASSO) analysis. Moreover, we explored in detail the tumor mutational signature, immune signature, and sensitivity to treatment of brain metastases in NSCLC. Finally, a nomogram was built using the rms package.

**Results:** First, 472 DEGs associated with brain metastases in NSCLC were obtained, which were closely associated with cancer-associated pathways. Interestingly, a RiskScore model was constructed using 11 genes from 472 DEGs, and the robustness was confirmed in GSE test, entire GSE, and TCGA datasets. Samples in the low RiskScore group had a higher gene mutation score and lower immunoinfiltration status. Moreover, we found that the patients in the low RiskScore group were more sensitive to the four chemotherapy drugs. In addition, the predictive nomogram model was able to effectively predict the outcome of patients through appropriate RiskScore stratification.

**Conclusion:** The prognostic RiskScore model we established has high prediction accuracy and survival prediction ability for brain metastases in NSCLC.

## 1 Introduction

Lung cancer mainly includes two main types: small cell lung cancer (SCLC) and non-small cell lung cancer (NSCLC). Lung cancer ranks as a leading cause of malignant tumor-induced death worldwide. Among them, NSCLC is more common, and its prevalence rate exceeds 80% (Grant et al., 2021). The brain is the most likely target for distant metastasis in NSCLC, and approximately 30% of the patients develop brain metastases during the cancer progression (Balasubramanian et al., 2020; Eguren-Santamaria et al., 2020). Previous studies reported that for most brain metastases, the cerebral hemisphere is the most easily invaded part, accounting for approximately 80%, and studies showed that the brainstem sites are the least likely to develop NSCLC metastases, occurring in less than 5% of the patients (D'Antonio et al., 2014; Wang et al., 2022). Regrettably, NSCLC with brain metastases, characterized by an extremely poor prognosis, is a major factor resulting in disability and death in advanced NSCLC. Brain metastases tend to seriously affect the cognitive function of patients, reduce the quality of life, and shorten the survival time (Teixeira Loiola de Alencar et al., 2021; Zhi et al., 2021). Currently, untreated median overall survival (OS) for patients with brain metastases is less than 3 months, and there are only few effective treatment options due to the presence of the blood–brain barrier (Dempke et al., 2015). Unfortunately, to date, strategies that can effectively predict the treatment effect after brain metastases in NSCLC are lacking.

Prognostic models developed using gene expression profiles of NSCLC have been reported previously. Kratz et al. (2012) developed a model for identifying patients who have small, node-negative lung tumors but at high risk of mortality. Currently, an integrated transcriptome and epigenome analysis identified 17 genes associated with NSCLC prognosis. These genes are associated with hypoxia response and NSCLC epigenetic modification (Chen et al., 2019). Moreover, a comprehensive study filtered six genes associated with an adenocarcinoma type of NSCLC based on integrated analysis and weighted gene co-expression network analysis (Xie and Xie, 2019). Although considerable work has been performed on NSCLC prognostic prediction, due to its complexity, predictive models for NSCLC brain metastases are unclear.

Here, in this work, we first identified differentially expression genes (DEGs) in NSCLC brain metastasis patients. Then, the genes significantly related to the overall survival of NSCLC patients were selected from the aforementioned genes based on the results of univariate Cox regression analysis. Finally, 11 prognostic genes of brain metastases in NSCLC were determined by multivariate Cox and LASSO regression analyses and used to build a RiskScore model. Moreover, we validated the efficiency of the model in detail through immune tumor microenvironment, drug sensitivity, survival, tumor mutation, and decision tree analyses.

## 2 Materials and methods

### 2.1 Lung cancer-related dataset download and quality control

To get a deeper insight into the mechanism of NSCLC patients with brain metastases, first, The Cancer Genome Atlas (TCGA) database was selected to download the transcriptomic expression, clinical survival, and characteristic information, including LUAD and LUSC, and 599 patients were obtained. The lung cancer microarray sequencing dataset with survival time was downloaded from the Gene Expression Omnibus (GEO) database, including GSE30219 (254 samples), GSE31210 (226 samples), GSE37745 (95 samples), and GSE50081 (176 samples) (Okayama et al., 2012; Rousseaux et al., 2013; Der et al., 2014; Goldmann et al., 2021). Moreover, a dataset of NSCLC with features of brain metastases, GSE200563 (Bader and Hogue, 2003), was also downloaded from the GEO database.

In order to obtain high-quality downstream analysis results, we performed quality control on the downloaded dataset. The quality control was performed using the following steps: remove samples without clinical follow-up information and samples without disease-free survival (DFS) time and status; convert gene names to uniform IDs; and merge the datasets and remove batch effects using the *removeBatchEffect* function of the limma package (R package) (Ritchie et al., 2015). Specifically, for GSE200563, we only considered two types of samples: primary lung cancer and metastatic lung cancer in the brain.

### 2.2 Identification of differentially expressed gene (DEG)

To identify the pathogenesis of brain metastases in NSCLC, we performed DEG analysis on patients with metastatic lung cancer in the brain and primary lung cancer of GSE200563 using the limma R package (Ritchie et al., 2015). The threshold for DEGs was set to |foldchang| > 1.2 and *p-value* < 0.05.

### 2.3 Function enrichment and protein–protein interaction (PPI) analyses

The WebGestaltR (R package) was selected to perform function enrichment analysis of DEGs, including Gene Ontology (GO) and Kyoto Encyclopedia of Genes and Genomes (KEGG) analyses (Liao et al., 2019). The threshold for significant difference of GO and KEGG terms was set to *p-value* < 0.05.

The Search Tool for Retrieval of Interacting Genes/Proteins (STRING) (https://string-db.org/, v11.0) was selected to perform PPI analysis on aforementioned DEGs, and Cytospace was used to visualize the PPI network (Kohl et al., 2011). Currently, the STRING database consists of 18,838 human proteins with 25, 914, 693 core network interactions. The highest confidence interaction score was set to 0.9, which reduces the number of false-positive interactions (Bozhilova et al., 2019). The molecular complex detection (MCODE) algorithm was used to perform network function module mining (Bader and Hogue, 2003). MCODE calculates accurate correlation levels and identifies essential PPI network modules.

### 2.4 Establishment of the prognostic model

The DEGs related to brain metastases in NSCLC were used to build the prognostic model. To avoid random assignment bias that could affect the stability of subsequent modeling, all samples in the GSE dataset were randomly grouped 100 times with playback beforehand, according to the ratio of the training set: validation set = 1:1. There were no significant differences between the two parts in DFS and status (Table 1). Then, we performed univariate Cox regression analysis on DEGs in the training dataset. Then, LASSO analysis was performed *via* glmnet (R package) to reduce the candidate prognostic genes (Zhang et al., 2022). Moreover, we used stepwise multivariate Cox regression analysis to identify the prognostic genes.

**TABLE 1:** Clinical information of the test and training datasets.

	Test (N = 377)	Training (N = 377)	*p*-value
			
DFS			
0	238 (63.1%)	250 (66.3%)	0.402
1	139 (36.9%)	127 (33.7%)	
DFS.time			
Mean (SD)	1580 (1360)	1720 (1420)	0.169
Median [min, max]	1490 [6.00, 7320]	1620 [7.00, 7680]	
Dataset			
GSE30219	129 (34.2%)	126 (33.4%)	0.938
GSE31210	109 (28.9%)	117 (31.0%)	
GSE37745	49 (13.0%)	47 (12.5%)	
GSE50081	90 (23.9%)	87 (23.1%)	

Significantly, we calculated each patient’s RiskScore using the following formula: RiskScore = Σβ*i* × Exp*i*, where Exp*i* refers to the gene expression level of the signature and β represents the Cox regression coefficient of the corresponding gene.

High- and low-risk groups of patients were divided based on the median threshold. The Kaplan–Meier method for prognostic analysis was employed for drawing survival curves, followed by studying the significant differences with the log-rank test. The time-dependent ROC curve showed survival of different risk groups, and survivalROC (R package) evaluated the prediction of the model (Heagerty and Zheng, 2005).

### 2.5 Tumor mutation analysis

Mutect2 was selected to conduct tumor mutation analysis (Prashant et al., 2021; Jin et al., 2022). First, genes showing a mutation frequency greater than 3 were obtained, and those with significantly high frequency mutations in each subtype were screened by Fisher’s test under *p-value* < 0.05. Next, the distribution of fraction altered, tumor mutation burden, number of segments, and homologous recombination defects of each subtype was studied.

### 2.6 Immune signature analysis

Immune signature analysis (ESTIMATE) was performed, including calculation of the immune score, immune infiltration score, and ESTIMATE score, for the purpose of elucidating differences in the patients’ immune microenvironment (Yoshihara et al., 2013). Based on the gene marker expression in immune cells, immune cell infiltration in patients could be analyzed (Becht et al., 2016). A total of 10 immune cells were scored by MCP-counter estimates and the single-sample gene set enrichment analysis (ssGSEA) algorithm (Becht et al., 2016; Charoentong et al., 2017) that counted 28 immune cells. ESTIMATE scored the overall immune microenvironment infiltration.

### 2.7 Immunotherapy/chemotherapy effect analysis

The effectiveness of immune mutation score (IMS) on predicting clinical responsiveness to immune checkpoint inhibitors (ICIs) was verified by applying the tumor immune dysfunction and exclusion (TIDE) algorithm (Jiang et al., 2018). Immune checkpoints obtained from the HisgAtlas database (Liu et al., 2017). Furthermore, we also performed treatment effect predictions for traditional chemotherapeutics using pRRophetic (R package), such as sorafenib, pyrimethamine, AKT inhibitor VIII, and embelin (Geeleher et al., 2014).

### 2.8 Pathway characteristic analysis of the RiskScore model

We performed GSEA pathway analysis on different RiskScore groups by using GSEA (R package) (Subramanian et al., 2005). The candidate background gene sets were obtained from the Hallmark database. A significant enrichment was defined when *FDR* < 0.05. Moreover, the correlations of different biological functions with RiskScore were also calculated.

### 2.9 Decision tree analysis to optimize the RiskScore prediction model

First, a decision tree based on age, sex, stage, T stage, N stage, and RiskScore of patients with NSCLC in TCGA cohort was generated. The univariate and multivariate Cox regression analyses of RiskScore and clinicopathological characteristics were performed. The reliability of RiskScore was evaluated with decision curve analysis (DCA).

## 3 Results

### 3.1 Transcriptional effects of brain metastases in NSCLC

**FIGURE 1:**
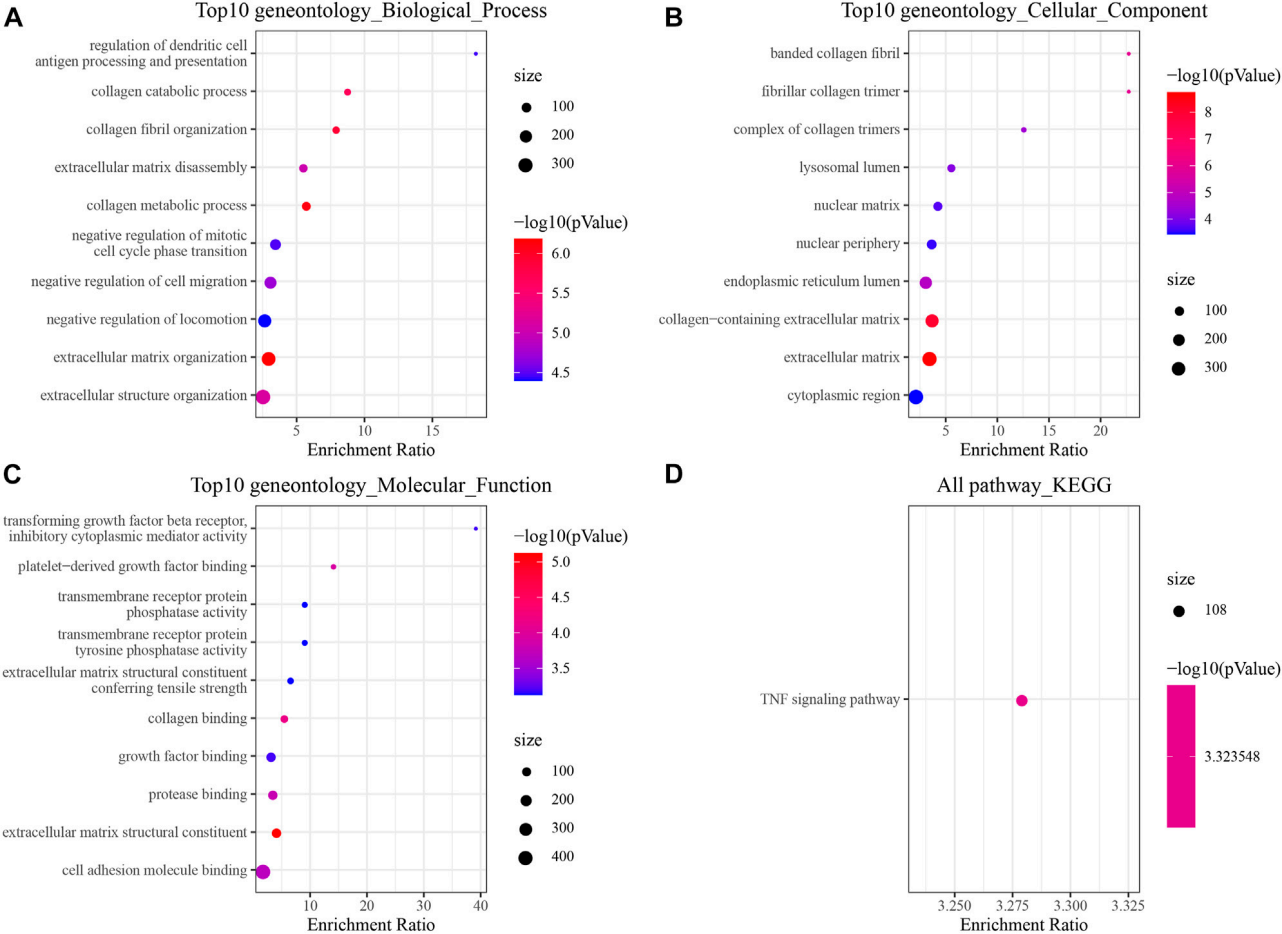
Changes in the transcriptome of brain metastases in NSCLC [GSE200563]. **(A)** Top 10 Gene Ontology (GO) terms at the biological process level. **(B)** Top 10 GO terms at the cellular component level. **(C)** Top 10 GO terms at the molecular function level. **(D)** Significantly enriched Kyoto Encyclopedia of Genes and Genomes (KEGG) pathway.

To gain insights into the pathogenic mechanism of brain metastases in NSCLC, we first performed differential analysis of the transcriptome data of brain metastases in NSCLC patients. Finally, 472 DEGs were obtained, of which 218 genes were upregulated in metastatic lung cancer in the brain and 254 genes were downregulated in metastatic lung cancer in the brain. The GO and KEGG pathway functional enrichment analyses were carried out. The results of GO analysis showed that DEGs related to metastatic lung cancer in the brain were involved in negative regulation of mitotic cell cycle phase transition and extracellular matrix disassembly (Figure 1A). Most DEGs involved in cellular components that make up the banded collagen fibril, nuclear matrix, and extracellular matrix (Figure 1B). Moreover, molecular functional analysis revealed that DEGs were involved in growth factor binding (Figure 1C). Interestingly, the KEGG pathway analysis observed that DEGs participated in the TNF signaling pathway (Figure 1D).

### 3.2 Functional network of DEGs related to brain metastases in NSCLC

**FIGURE 2:**
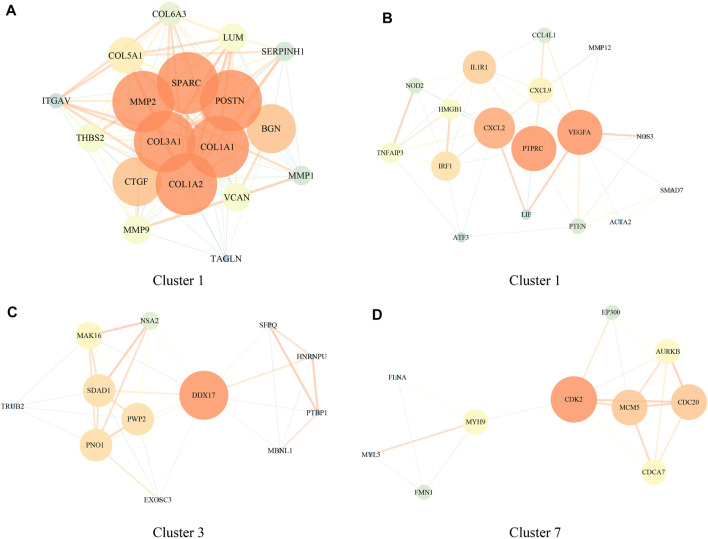
Protein–protein interaction (PPI) network of genes involved in regulating brain metastases in NSCLC [GSE200563]. **(A–D)** PPI network in clusters 1, 2, 3, and 7.

To understand the role of DEGs in brain metastases in NSCLC, we further performed PPI analysis using STRING, and the results showed that these DEGs had four closely related functional networks. Cluster 1 was closely related to bladder cancer, ECM–receptor interaction, proteoglycans in cancer, and PI3K-Akt signaling pathway (Figure 2A and Supplementary Figure S1). Cluster 2 was involved in regulating the NF-κB signaling pathway and TNF signaling pathway (Figure 2B and Supplementary Figure S2). Cluster 3 was related to the regulation of RNA splicing (Figure 2C and Supplementary Figure S3). Cluster 7 was associated with cell cycle and FOXO signaling pathway (Figure 2D and Supplementary Figure S4).

### 3.3 Identification of prognostic genes associated with brain metastases in NSCLC

**FIGURE 3:**
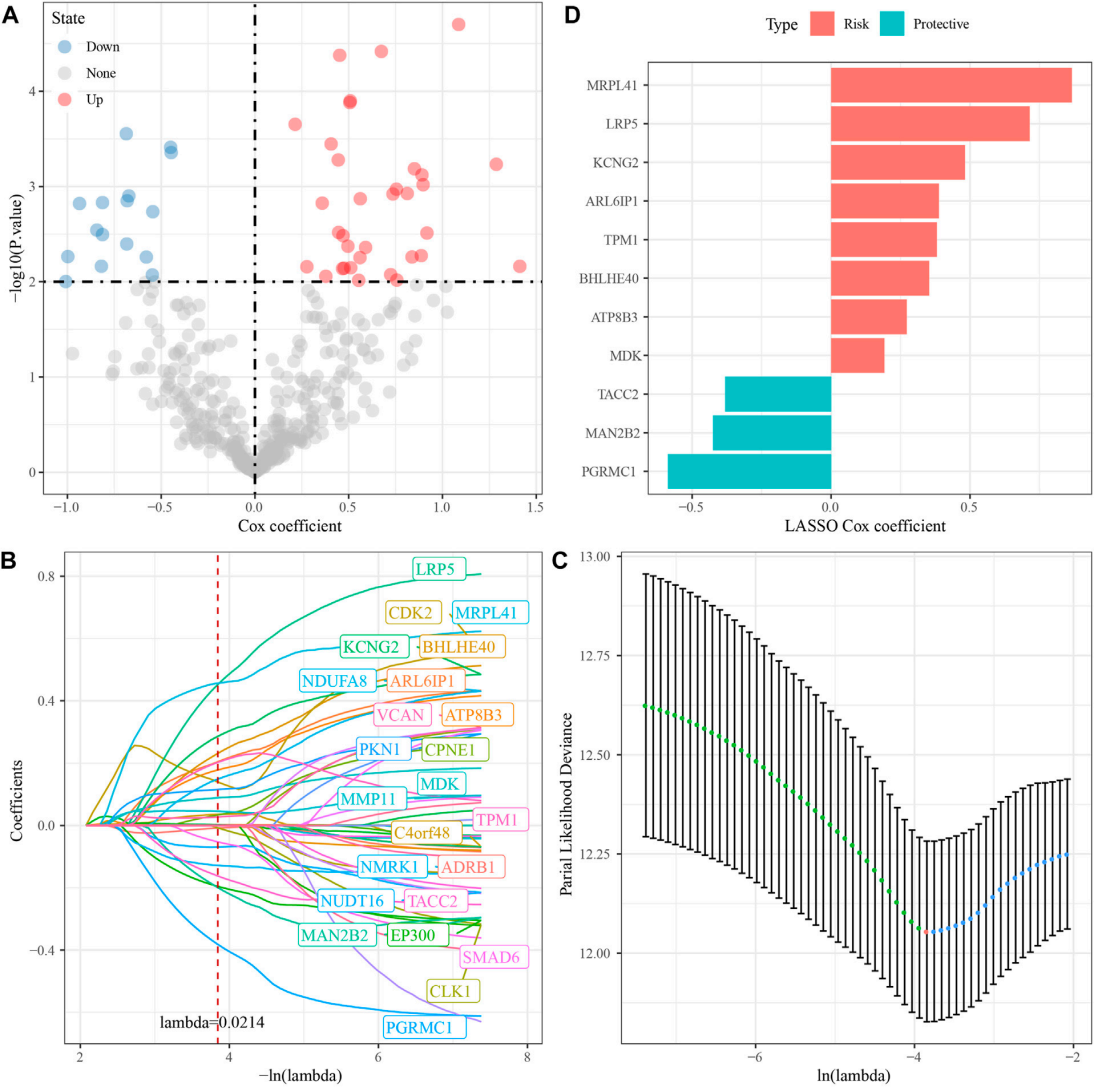
Identification of RiskScore model prognostic genes [GSE200563]. **(A)** Totally 11 promising candidates were identified through the survival analysis of the genes of the blue module. **(B)** Trajectory of candidate genes changes as lambda changes. **(C)** Confidence intervals for different lambda values. **(D)** Distribution of LASSO coefficients of the prognostic gene signature.

Briefly, the GSE dataset was first divided into two parts randomly according to the ratio of training: test = 1:1; then, a univariate Cox regression analysis was performed on DEGs in the training dataset; and a total of 50 prognostic factors were identified (*p-*value < 0.01), which contained 34 “risk” genes and 16 “protective” genes (Figure 3A). Then, the LASSO algorithm was used to further narrow down the gene range, and the change trajectory of each DEG is shown in Figure 3B. When lambda = 0.0214, the model reached the optimum, so we selected 24 genes as the next target gene (Figure 3C). Stepwise multivariate regression analysis was performed on the genes screened by LASSO algorithm, and finally, 11 prognostic genes associated with brain metastases in NSCLC were selected (Figure 3D).

### 3.4 Construction of the prognostic RiskScore model

**FIGURE 4:**
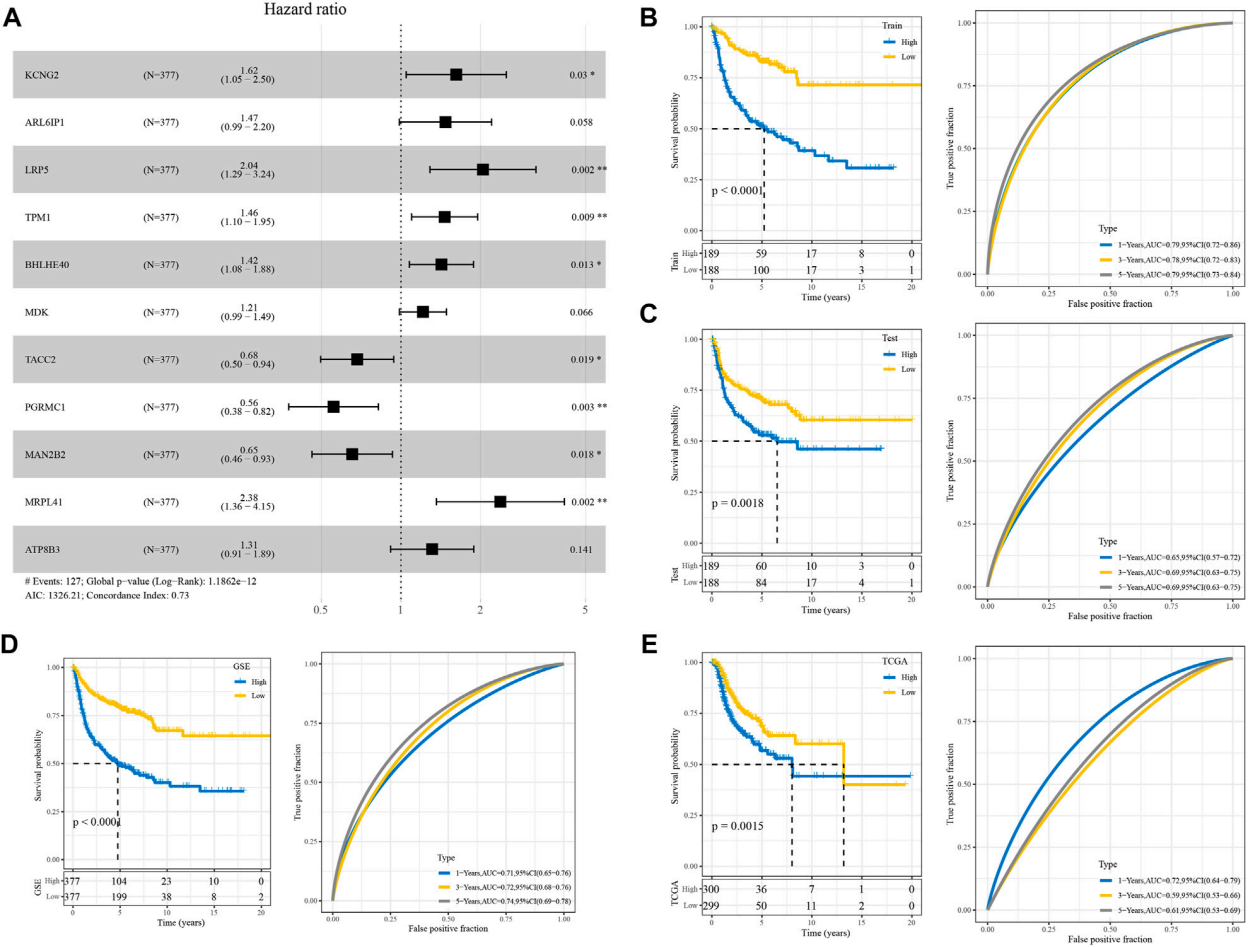
Validation of the RiskScore model. **(A)** Forest diagram of multivariate Cox analysis of the model genes. **(B)** ROC and KM curves of RiskScore using GSE training data. **(C)** Verifying ROC curve and KM curve of RiskScore in “my data queue” in GSE. **(D)** ROC and KM curves of RiskScore in the GSE cohort. **(E)** ROC and KM curves of RiskScore in TCGA cohort.

The forest plot showed that among 11 prognostic genes, eight genes led to poorer prognosis and the others were related to better prognosis (Figure 4A). We constructed a prognostic model using the following formula: RiskScore = Σβ*i* × Exp*i*. We divided the samples into two groups of high and low risk, with the median value of RiskScore as a cutoff, and drew the KM curve, and the results showed that there were very significant differences between the different groups (Figure 4B). The same analysis was performed in GSE and TCGA cohorts which showed poor prognosis in the high RiskScore group (Figures 4C–E).

### 3.5 The mutation signature between RiskScore groupings

To explore differences in genomic alterations between different RiskScore groups in TCGA cohort, we performed tumor mutation analysis. With a selection threshold of *p*-value < 0.05, a total of 263 genes showing significantly high frequency mutations were screened between different RiskScore groups (Supplementary Table S1). The mutational signatures of the top 20 genes are shown in Supplementary Figure S5A. Distributions of the number of segments, fraction altered, tumor mutation burden, and homologous recombination defects among subtypes were compared; however, these mutational signatures did not differ significantly across the different RiskScore groups (Supplementary Figure S5B).

### 3.6 The immune signature between RiskScore groupings

**FIGURE 5:**
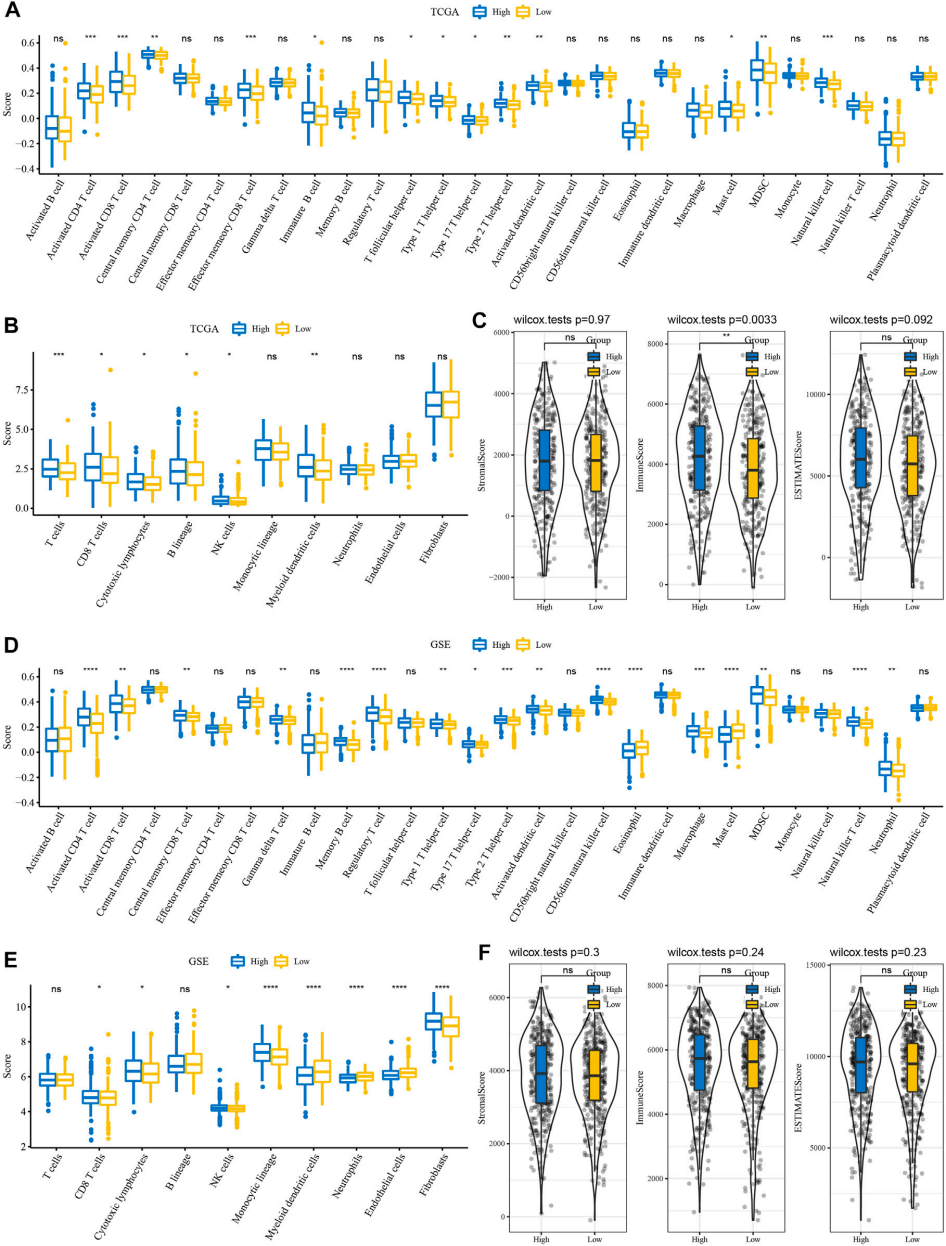
Immune features between RiskScore groupings. **(A)** ssGSEA evaluated the subtypes of 28 immune cell scores in TCGA cohort. **(B)** MCP-counter evaluated subtype comparison of 10 immune cell scores in TCGA cohort. **(C)** ESTIMATE subtype comparison of StromalScore, ImmuneScore, and ESTIMATEScore in TCGA cohort. **(D)** Subtype comparison of 28 immune cell scores assessed in the GSE cohort with ssGSEA. **(E)** Subtype comparison of 10 immune cell scores assessed in the GSE cohort with MCP-counter. **(F)** Subtype comparison of StromalScore, ImmuneScore, and ESTIMATEScore in the GSE cohort with ESTIMATE.

Immune cell infiltration in TCGA and GSE cohort patients were analyzed using gene marker expression in immune cells. The results of ssGSEA showed that among the 28 types of immune cells, the immune score of the high-risk group was also high among the RiskScore groups (Figure 5A). Interestingly, the results of MCP-counter estimates suggested that even among the high RiskScore group, the immune score was higher (Figure 5B). The results of ESTIMATE, including stromal score, immune score, and ESTIMATE, were in line with those of the ssGSEA and MCP-counter (Figure 5C). Moreover, it was similar to TCGA cohort results, while the GSE cohort showed similar trends (Figures 5E,F).

### 3.7 Differences in immunotherapy/chemotherapy between RiskScore groupings

**FIGURE 6:**
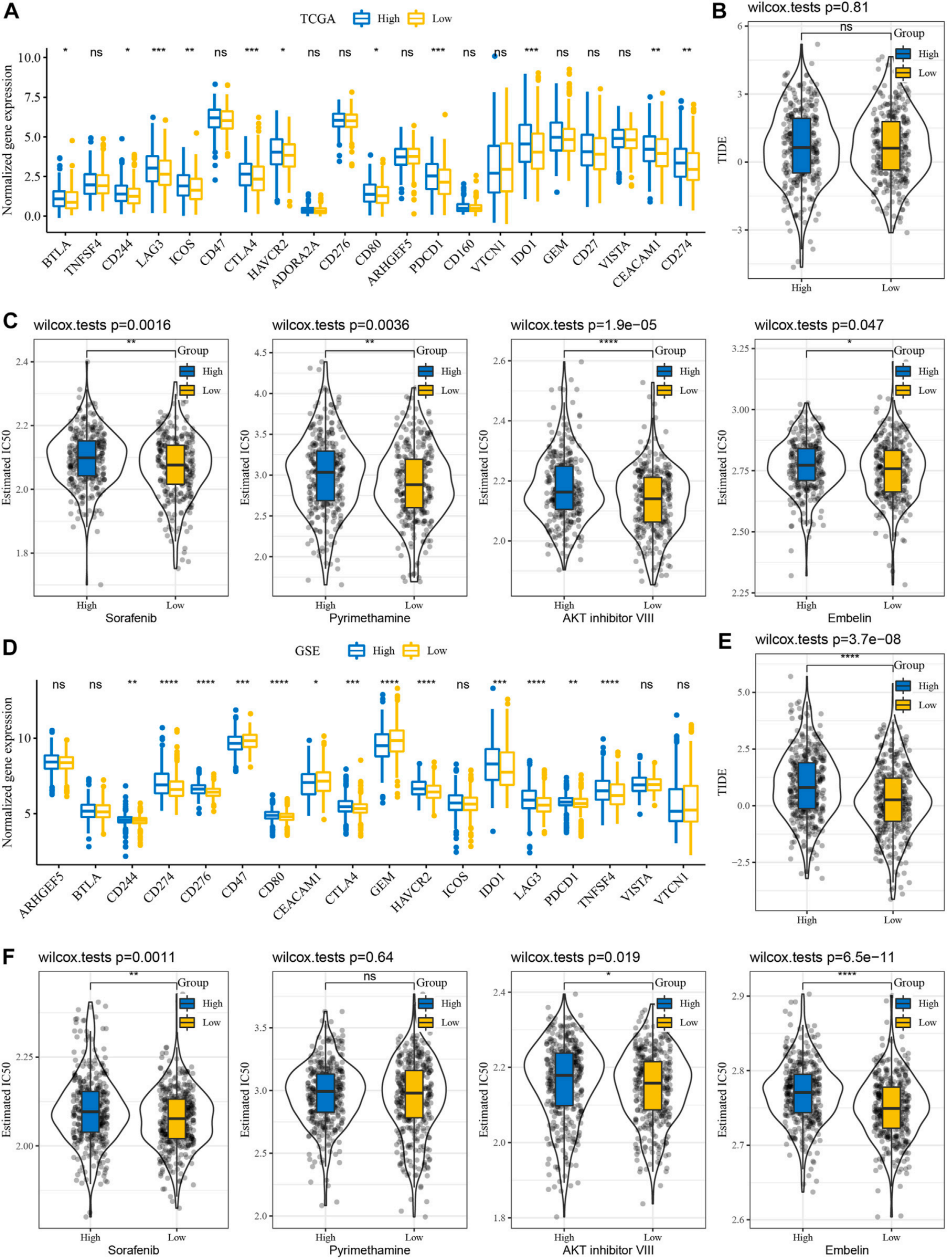
Immunotherapy/chemotherapy sensitivity analysis. **(A)** Immunological checkpoint of differential expression between different groups in TCGA cohort. **(B)** Difference in TIDE analysis results among different groups in TCGA queue. **(C)** Box plots of the estimated IC_50_ for sorafenib, pyrimethamine, Akt inhibitor VIII, and embelin in TCGA cohort. **(D)** Differentially expressed immune checkpoints between different subgroups in the GSE cohort. **(E)** Differences in TIDE analysis results among different groups in GSE queues. **(F)** Box plots of the estimated IC_50_ for sorafenib, pyrimethamine, Akt inhibitor VIII, and embelin in GSE.

We further analyzed whether there exist differences in response to immunotherapy/chemotherapy between different RiskScore groups. First, the expression level of immune checkpoints differed between RiskScore groupings (Figure 6A). The results showed that only some immune checkpoints were differentially expressed between RiskScore groupings, like LAG3 and CD244 (Figure 6A). We observed no difference in TIDE scores between high and low RiskScore groups in TCGA cohort (Figure 6B). It was found that in TCGA cohort, the low RiskScore group was more sensitive to these four drugs: sorafenib, pyrimethamine, Akt inhibitor VIII, and embelin (Figure 6C). Moreover, in the analysis of the GSE cohort, the expression of immune checkpoints was significantly different (Figure 6D). Interestingly, the TIDE score was higher in the high RiskScore groups (Figure 6E). Drug analysis showed that the low RiskScore group was more sensitive to chemotherapy drugs (Figure 6F).

### 3.8 The pathway signature between RiskScore groupings

**FIGURE 7:**
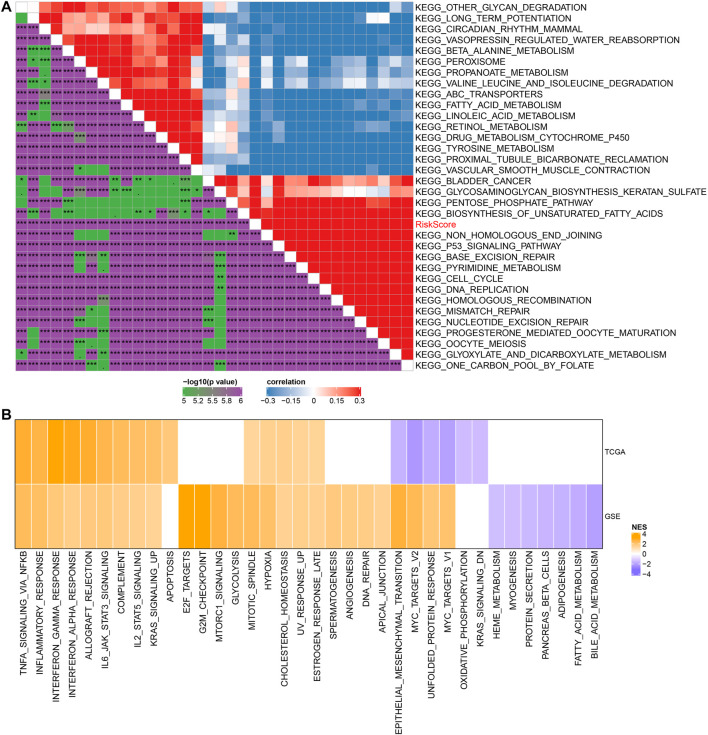
Relationship between RiskScore and KEGG pathways. **(A)** Heat map showing the correlation between RiskScore and KEGG pathways. **(B)** Heat map demonstrating normalized enrichment scores of Hallmark pathways calculated by comparing high RiskScore with low RiskScore.

To observe the relationship between RiskScore and biological function, we performed functional enrichment analysis and correlation analysis on NSCLC samples in the GSE cohort. The results showed that these pathways were positively correlated with RiskScore of the samples, and these pathways were mainly tumor-related pathways, such as p53 signaling pathway and DNA replication (Figure 7A). In addition, GSEA results showed that in TCGA cohort, compared with the low RiskScore group, 15 pathways were activated in the high RiskScore group and 26 pathways were activated and seven pathways were inhibited in the GSE cohort (Figure 7B). The active pathways in the high RiskScore group were mainly tumor-correlated pathways, such as KRAS_SIGNALING_UP and HYPOXIA, IL6_JAK_STAT3_SIGNALING, and TNFA_SIGNALING_VIA_NFKB*.* (Figure 7B).

### 3.9 Combining clinicopathological features to improve the prognosis model and survival prediction

**FIGURE 8:**
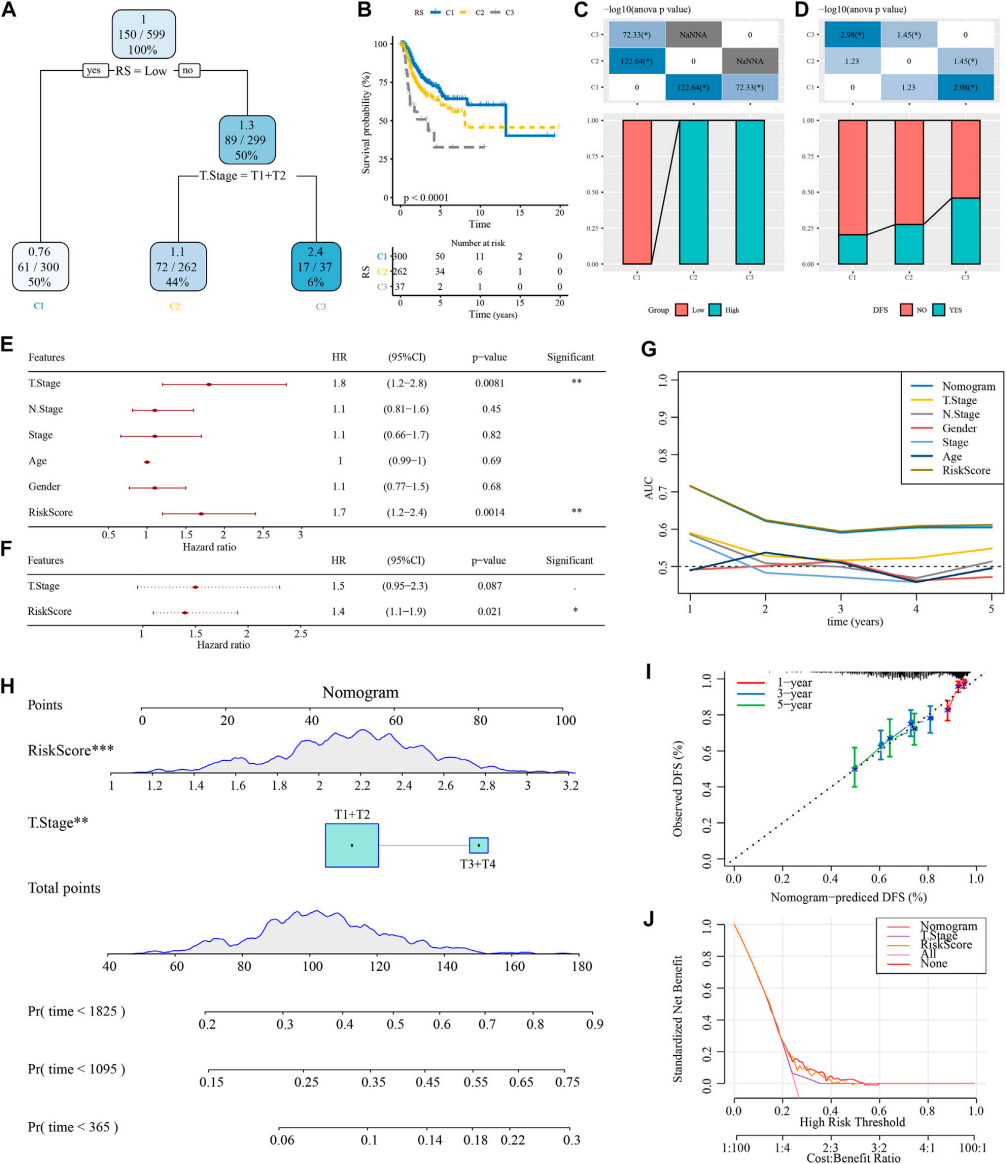
Optimization of the RiskScore model. **(A)** Patients with full-scale annotations including RiskScore, stage, gender, and age were used to build a survival decision tree to optimize risk stratification. **(B)** Significant differences in overall survival were observed among the three risk subgroups. **(C**, **D)** Comparative analysis among the different groups. **(E**, **F)** Univariate and multivariate Cox analysis of RiskScore and clinicopathological features. **(G)** Compared with other clinicopathological features, the nomogram exhibited the most powerful capacity for survival prediction. **(H)** Alignment diagram showing the influence of different factors on the prediction results; the top panel shows scores, the middle panel shows different factors, and the bottom panel shows predictive efficiency. **(I)** Calibration curves of the 1, 3, and 5 years of the line chart. **(J)** Decision curve of the line graph.

A decision tree based on T stage, N stage, age, sex, stage, and RiskScore of patients with NSCLC in TCGA cohort was developed, but only RiskScore and T stage remained, and we categorized three risk subgroups (Figure 8A) with significant overall survival differences (Figure 8B). The risk subgroups C2 and C3 contained high RiskScore patients, while the “C1” group contained low RiskScore patients (Figure 8C). Patients in different risk subgroups had different survival statuses (Figure 8D). Univariate and multivariate Cox regression analyses of RiskScore and clinicopathological features validated RiskScore as the most significant factor for prognosis (Figures 8E,F). To quantify the risk assessment and patients’ survival, other clinicopathological features were combined with RiskScore to build a nomogram (Figure 8H). RiskScore showed the greatest influence on the survival prediction. The model prediction accuracy (Figure 8I) and reliability were evaluated using RiskScore and DCA, respectively. Compared with the extreme curves, both RiskScore and nomogram had significantly higher benefits. Furthermore, RiskScore and nomogram showed the strongest survival among other clinicopathological features (Figures 8G,J).

## 4 Discussion

Brain metastases are a common complication of NSCLC, with an incidence of more than 30% and often extremely distressing, and most seriously, a very short survival period (Goldmann et al., 2021; Wang et al., 2022). Therefore, it becomes important to develop a stable prognostic indicator. Here, we developed a RiskScore prognostic model containing 11 prognostic genes for predicting the prognosis of brain metastases in NSCLC based on data from TCGA and combining clinicopathological features to further improve the prognostic model and survival prediction. Interestingly, we found that in TCGA and GSE cohorts, the high RiskScore groups had a poorer prognosis, while the low RiskScore groups had a better prognosis. The results of function enrichment analysis suggested that the expression changes of the p53 signaling pathway-related genes were the key to the different RiskScore groups. Moreover, the differences in the immune profile were also a factor leading to different RiskScore groups, which is reported by many studies (Chen et al., 2019; Xie and Xie, 2019).

We noticed that after the occurrence of brain metastasis, the transcriptional expression profile of NSCLC patients also changed greatly, and we obtained a total of 472 DEGs. The KEGG pathway analysis showed that the disorder of the TNF signaling pathway was one of the causes of the emergency of brain metastases. Many studies showed that TNF and its receptors were widely expressed in NSCLC, and the mechanism of action was very complex (Gong et al., 2021). The high expression of TNF in NSCLC patients determined its poor prognosis (Gong et al., 2021). Moreover, PPI analysis suggested that the DEGs were related to the PI3K-Akt signaling pathway. Zhou et al. (2021) reported the abnormal expression of the PI3K-Akt signaling pathway, which caused tumor growth and metastasis in NSCLC.

Through univariate Cox and LASSO regression analyses, we identified 11 prognostic genes as RiskScore model building genes: *MRPL41*, *LRP5*, *KCNG2*, *ARL6IP1*, *TPM1*, *BHLHE40*, *ATP8B3*, *MDK*, *TACC2*, *MAN2B2*, and *PGRMC1*. *MRPL41* encodes a mitochondrial protein, and it can arrest the cell cycle and induce apoptosis (Goldschmidt-Reisin et al., 1998; Yoo et al., 2005). The study reported that the proliferation rate of NCI-H211 cells decreased after overexpression of MRPL41 (Yoo et al., 2005). *LRP5* is associated with activation of the Wnt signaling pathway, and in NSCLC, LRP5 polymorphisms play a role in NSCLC susceptibility (Williams and Insogna, 2009; Wang et al., 2016). In one study, *PGRMC1* was found to induce erlotinib resistance, triggering crosstalk of the Wnt/β-catenin and NF-κB pathways in lung adenocarcinoma cells (Ma and Hottiger, 2016). As early as 2006, *TACC3* was reported as a prognostic marker for NSCLC (Jung et al., 2006). In two independent epidemiological genetic characterization surveys in all locations, the incidence of *FGFR–TACC* gene fusions was extremely high in NSCLC (Zheng et al., 2020). Survival analysis was an excellent method to verify the validity of model predictions (Han et al., 2021; Jiang et al., 2021). In our survival analysis, we found that in the low RiskScore group, survival was significantly longer. Therefore, we have reason to believe the validity of our RiskScore model.

We noticed that there were differences in the tumor immune microenvironment between different RiskScore groups, which was manifested in the higher immune score in the high group. Significantly, we found the immune infiltration of CD4^+^ T cell and CD8^+^ T cell. In the high RiskScore group, it was higher than that in the low RiskScore group. Different types of immune cells played different roles in the process of anti-tumor and tumor immune escape. The growth, invasion, and metastasis of tumors were all related to the immune microenvironment (Chen et al., 2021; Mao et al., 2021; Qiao et al., 2021). Moreover, the results of sensitivity analysis to immunotherapy suggested that low RiskScore patients were more sensitive to chemotherapy drugs. Based on the aforementioned results, we speculated that in this case, despite showing substantial immune cell infiltration, they may not be able to penetrate the tumor parenchyma efficiently to eliminate tumor cells. Therefore, it was not surprising that high RiskScore groups tend to have poorer outcomes.

Moreover, we combined RiskScore with clinicopathological features using decision tree models to further improve prognostic models and survival predictions. In conclusion, our results demonstrated that our RiskScore model has good predictive power for the prognosis of brain metastases in NSCLC.

## 5 Conclusion

In this work, the 472 DEGs related to brain metastases in NSCLC were obtained. Significantly, based on brain metastasis-related genes, we constructed the RiskScore clinical prognosis model which showed strong robustness, is independent of clinicopathological characteristics, and had stable predictive performance in independent datasets.

## Data Availability

The original contributions presented in the study are included in the article/Supplementary Material; further inquiries can be directed to the corresponding author.

## References

[B1] Bader G. D. Hogue C. W. (2003). An automated method for finding molecular complexes in large protein interaction networks. BMC Bioinforma. 4, 2. 10.1186/1471-2105-4-2 PMC149346 12525261

[B2] Balasubramanian S. K. Sharma M. Venur V. A. Schmitt P. Kotecha R. Chao S. T. (2020). Impact of EGFR mutation and ALK rearrangement on the outcomes of non-small cell lung cancer patients with brain metastasis. Neuro-oncology. 22 (2), 267–277. 10.1093/neuonc/noz155 31648302 PMC7442419

[B3] Becht E. Giraldo N. A. Lacroix L. Buttard B. Elarouci N. Petitprez F. (2016). Estimating the population abundance of tissue-infiltrating immune and stromal cell populations using gene expression. Genome Biol. 17 (1), 218. 10.1186/s13059-016-1070-5 27765066 PMC5073889

[B4] Bozhilova L. V. Whitmore A. V. Wray J. Reinert G. Deane C. M. (2019). Measuring rank robustness in scored protein interaction networks. BMC Bioinforma. 20 (1), 446. 10.1186/s12859-019-3036-6 PMC6714100 31462221

[B5] Charoentong P. Finotello F. Angelova M. Mayer C. Efremova M. Rieder D. (2017). Pan-cancer immunogenomic analyses reveal genotype-immunophenotype relationships and predictors of response to checkpoint blockade. Cell. Rep. 18 (1), 248–262. 10.1016/j.celrep.2016.12.019 28052254

[B6] Chen X. Xu R. He D. Zhang Y. Chen H. Zhu Y. (2021). CD8(+) T effector and immune checkpoint signatures predict prognosis and responsiveness to immunotherapy in bladder cancer. Oncogene 40 (43), 6223–6234. 10.1038/s41388-021-02019-6 34552192

[B7] Chen Y. L. Zhang Y. Wang J. Chen N. Fang W. Zhong J. (2019). A 17 gene panel for non-small-cell lung cancer prognosis identified through integrative epigenomic-transcriptomic analyses of hypoxia-induced epithelial-mesenchymal transition. Mol. Oncol. 13 (7), 1490–1502. 10.1002/1878-0261.12491 30973670 PMC6599842

[B8] D'Antonio C. Passaro A. Gori B. Del Signore E. Migliorino M. R. Ricciardi S. (2014). Bone and brain metastasis in lung cancer: Recent advances in therapeutic strategies. Ther. Adv. Med. Oncol. 6 (3), 101–114. 10.1177/1758834014521110 24790650 PMC3987652

[B9] Dempke W. C. Edvardsen K. Lu S. Reinmuth N. Reck M. Inoue A. (2015). Brain metastases in NSCLC - are TKIs changing the treatment strategy? Anticancer Res. 35 (11), 5797–5806. 26504000

[B10] Der S. D. Sykes J. Pintilie M. Zhu C. Q. Strumpf D. Liu N. (2014). Validation of a histology-independent prognostic gene signature for early-stage, non-small-cell lung cancer including stage IA patients. J. Thorac. Oncol. official Publ. Int. Assoc. Study Lung Cancer 9 (1), 59–64. 10.1097/JTO.0000000000000042 24305008

[B11] Eguren-Santamaria I. Sanmamed M. F. Goldberg S. B. Kluger H. M. Idoate M. A. Lu B. Y. (2020). PD-1/PD-L1 blockers in NSCLC brain metastases: Challenging paradigms and clinical practice. Clin. cancer Res. official J. Am. Assoc. Cancer Res. 26 (16), 4186–4197. 10.1158/1078-0432.CCR-20-0798 32354698

[B12] Geeleher P. Cox N. Huang R. S. (2014). pRRophetic: an R package for prediction of clinical chemotherapeutic response from tumor gene expression levels. PloS one 9 (9), e107468. 10.1371/journal.pone.0107468 25229481 PMC4167990

[B13] Goldmann T. Marwitz S. Nitschkowski D. Krupar R. Backman M. Elfving H. (2021). PD-L1 amplification is associated with an immune cell rich phenotype in squamous cell cancer of the lung. Cancer Immunol. Immunother. 70 (9), 2577–2587. 10.1007/s00262-020-02825-z 33576873 PMC8360842

[B14] Goldschmidt-Reisin S. Kitakawa M. Herfurth E. Wittmann-Liebold B. Grohmann L. Graack H. R. (1998). Mammalian mitochondrial ribosomal proteins. N-terminal amino acid sequencing, characterization, and identification of corresponding gene sequences. J. Biol. Chem. 273 (52), 34828–34836. 10.1074/jbc.273.52.34828 9857009

[B15] Gong K. Guo G. Beckley N. Zhang Y. Yang X. Sharma M. (2021). Tumor necrosis factor in lung cancer: Complex roles in biology and resistance to treatment. Neoplasia (New York, NY) 23 (2), 189–196. 10.1016/j.neo.2020.12.006 PMC7773536 33373873

[B16] Grant M. J. Herbst R. S. Goldberg S. B. (2021). Selecting the optimal immunotherapy regimen in driver-negative metastatic NSCLC. Nat. Rev. Clin. Oncol. 18 (10), 625–644. 10.1038/s41571-021-00520-1 34168333

[B17] Han K. Wang J. Qian K. Zhao T. Liu X. Zhang Y. (2021). Construction of a prognostic model for non-small-cell lung cancer based on ferroptosis-related genes. Biosci. Rep. 41 (5), BSR20210527. 10.1042/BSR20210527 33988228 PMC8170652

[B18] Heagerty P. J. Zheng Y. (2005). Survival model predictive accuracy and ROC curves. Biometrics 61 (1), 92–105. 10.1111/j.0006-341X.2005.030814.x 15737082

[B19] Jiang P. Gu S. Pan D. Fu J. Sahu A. Hu X. (2018). Signatures of T cell dysfunction and exclusion predict cancer immunotherapy response. Nat. Med. 24 (10), 1550–1558. 10.1038/s41591-018-0136-1 30127393 PMC6487502

[B20] Jiang X. Yan Q. Xie L. Xu S. Jiang K. Huang J. (2021). Construction and validation of a ferroptosis-related prognostic model for gastric cancer. J. Oncol. 2021, 6635526. 10.1155/2021/6635526 33727924 PMC7937463

[B21] Jin J. Chen Z. Liu J. Du H. Zhang G. (2022). Towards an accurate and robust analysis pipeline for somatic mutation calling. Front. Genet. 13, 979928. 10.3389/fgene.2022.979928 36457740 PMC9705725

[B22] Jung C. K. Jung J. H. Park G. S. Lee A. Kang C. S. Lee K. Y. (2006). Expression of transforming acidic coiled-coil containing protein 3 is a novel independent prognostic marker in non-small cell lung cancer. Pathol. Int. 56 (9), 503–509. 10.1111/j.1440-1827.2006.01998.x 16930330

[B23] Kohl M. Wiese S. Warscheid B. (2011). Cytoscape: Software for visualization and analysis of biological networks. Methods Mol. Biol. Clift. NJ) 696, 291–303. 10.1007/978-1-60761-987-1_18 21063955

[B24] Kratz J. R. Van den Eeden S. K. He J. Jablons D. M. Mann M. J. (2012). A prognostic assay to identify patients at high risk of mortality despite small, node-negative lung tumors. Jama 308 (16), 1629–1631. 10.1001/jama.2012.13551 23093159

[B25] Liao Y. Wang J. Jaehnig E. J. Shi Z. Zhang B. (2019). WebGestalt 2019: Gene set analysis toolkit with revamped UIs and APIs. Nucleic acids Res. 47 (W1), W199–W205. 10.1093/nar/gkz401 31114916 PMC6602449

[B26] Liu Y. He M. Wang D. Diao L. Liu J. Tang L. (2017). HisgAtlas 1.0: A human immunosuppression gene database. Database (Oxford) 2017, bax094. 10.1093/database/bax094 31725860 PMC7243927

[B27] Ma B. Hottiger M. O. (2016). Crosstalk between wnt/β-catenin and NF-κB signaling pathway during inflammation. Front. Immunol. 7, 378. 10.3389/fimmu.2016.00378 27713747 PMC5031610

[B28] Mao X. Xu J. Wang W. Liang C. Hua J. Liu J. (2021). Crosstalk between cancer-associated fibroblasts and immune cells in the tumor microenvironment: New findings and future perspectives. Mol. cancer 20 (1), 131. 10.1186/s12943-021-01428-1 34635121 PMC8504100

[B29] Okayama H. Kohno T. Ishii Y. Shimada Y. Shiraishi K. Iwakawa R. (2012). Identification of genes upregulated in ALK-positive and EGFR/KRAS/ALK-negative lung adenocarcinomas. Cancer Res. 72 (1), 100–111. 10.1158/0008-5472.CAN-11-1403 22080568

[B30] Prashant N. M. Liu H. Dillard C. Ibeawuchi H. Alsaeedy T. Chan H. (2021). Improved SNV discovery in barcode-stratified scRNA-seq alignments. Genes. 12 (10), 1558. 10.3390/genes12101558 34680953 PMC8535975

[B31] Qiao M. Jiang T. Liu X. Mao S. Zhou F. Li X. (2021). Immune checkpoint inhibitors in EGFR-mutated NSCLC: Dusk or dawn? Journal of thoracic oncology. official Publ. Int. Assoc. Study Lung Cancer 16 (8), 1267–1288. 10.1016/j.jtho.2021.04.003 33915248

[B32] Ritchie M. E. Phipson B. Wu D. Hu Y. Law C. W. Shi W. (2015). Limma powers differential expression analyses for RNA-sequencing and microarray studies. Nucleic acids Res. 43 (7), e47. 10.1093/nar/gkv007 25605792 PMC4402510

[B33] Rousseaux S. Debernardi A. Jacquiau B. Vitte A. L. Vesin A. Nagy-Mignotte H. (2013). Ectopic activation of germline and placental genes identifies aggressive metastasis-prone lung cancers. Sci. Transl. Med. 5 (186), 186ra66. 10.1126/scitranslmed.3005723 PMC4818008 23698379

[B34] Subramanian A. Tamayo P. Mootha V. K. Mukherjee S. Ebert B. L. Gillette M. A. (2005). Gene set enrichment analysis: A knowledge-based approach for interpreting genome-wide expression profiles. Proc. Natl. Acad. Sci. U. S. A. 102 (43), 15545–15550. 10.1073/pnas.0506580102 16199517 PMC1239896

[B35] Teixeira Loiola de Alencar V. Guedes Camandaroba M. P. Pirolli R. Fogassa C. A. Z. Cordeiro de Lima V. C. (2021). Immunotherapy as single treatment for patients with NSCLC with brain metastases: A systematic review and meta-analysis-the META-L-BRAIN study. J. Thorac. Oncol. official Publ. Int. Assoc. Study Lung Cancer 16 (8), 1379–1391. 10.1016/j.jtho.2021.04.014 33964398

[B36] Wang B. Chen S. Xiao H. Zhang J. Liang D. Shan J. (2022). Analysis of risk factors and gene mutation characteristics of different metastatic sites of lung cancer. Cancer Med. 11 (1), 268–280. 10.1002/cam4.4424 34799997 PMC8704150

[B37] Wang Y. Zhang Y. Fang M. Bao W. Deng D. (2016). Two novel susceptibility loci for non-small cell lung cancer map to low-density lipoprotein receptor-related protein 5. Oncol. Lett. 12 (4), 2307–2318. 10.3892/ol.2016.4954 27698794 PMC5038383

[B38] Williams B. O. Insogna K. L. (2009). Where wnts went: The exploding field of Lrp5 and Lrp6 signaling in bone. J. bone mineral Res. official J. Am. Soc. Bone Mineral Res. 24 (2), 171–178. 10.1359/jbmr.081235 PMC3276354 19072724

[B39] Xie H. Xie C. (2019). A six-gene signature predicts survival of adenocarcinoma type of non-small-cell lung cancer patients: A comprehensive study based on integrated analysis and weighted gene coexpression network. BioMed Res. Int. 2019, 4250613. 10.1155/2019/4250613 31886214 PMC6925693

[B40] Yoo Y. A. Kim M. J. Park J. K. Chung Y. M. Lee J. H. Chi S. G. (2005). Mitochondrial ribosomal protein L41 suppresses cell growth in association with p53 and p27Kip1. Mol. Cell. Biol. 25 (15), 6603–6616. 10.1128/MCB.25.15.6603-6616.2005 16024796 PMC1190350

[B41] Yoshihara K. Shahmoradgoli M. Martínez E. Vegesna R. Kim H. Torres-Garcia W. (2013). Inferring tumour purity and stromal and immune cell admixture from expression data. Nat. Commun. 4, 2612. 10.1038/ncomms3612 24113773 PMC3826632

[B42] Zhang Z. Zeng X. Wu Y. Liu Y. Zhang X. Song Z. (2022). Cuproptosis-related risk score predicts prognosis and characterizes the tumor microenvironment in hepatocellular carcinoma. Front. Immunol. 13, 925618. 10.3389/fimmu.2022.925618 35898502 PMC9311491

[B43] Zheng R. Yin Z. Alhatem A. Lyle D. You B. Jiang A. S. (2020). Epidemiologic features of NSCLC gene alterations in hispanic patients from Puerto Rico. Cancers 12 (12), 3492. 10.3390/cancers12123492 33255238 PMC7761356

[B44] Zhi X. Luo J. Li W. Wang J. Wang Y. Cai Y. (2021). Case report: Osimertinib followed by osimertinib plus bevacizumab, personalized treatment strategy for a lung cancer patient with a novel EGFR exon 20 insertion D770_N771insGT and multiple brain metastases. Front. Oncol. 11, 733276. 10.3389/fonc.2021.733276 34760695 PMC8573166

[B45] Zhou J. Peng Y. Gao Y. C. Chen T. Y. Li P. C. Xu K. (2021). Targeting DNAJC19 overcomes tumor growth and lung metastasis in NSCLC by regulating PI3K/AKT signaling. Cancer Cell. Int. 21 (1), 338. 10.1186/s12935-021-02054-z 34217321 PMC8254338

