# Supplementary material for: An effective prognostic model for assessing prognosis of non-small cell lung cancer with brain metastases
Source: Front Genet. 2023 Apr 13;14:1156322. doi: 10.3389/fgene.2023.1156322 (PMC10143500; doi:10.3389/fgene.2023.1156322)
Supplement: Supplementary file 1 [file DataSheet1.zip › Figure S2.pdf]

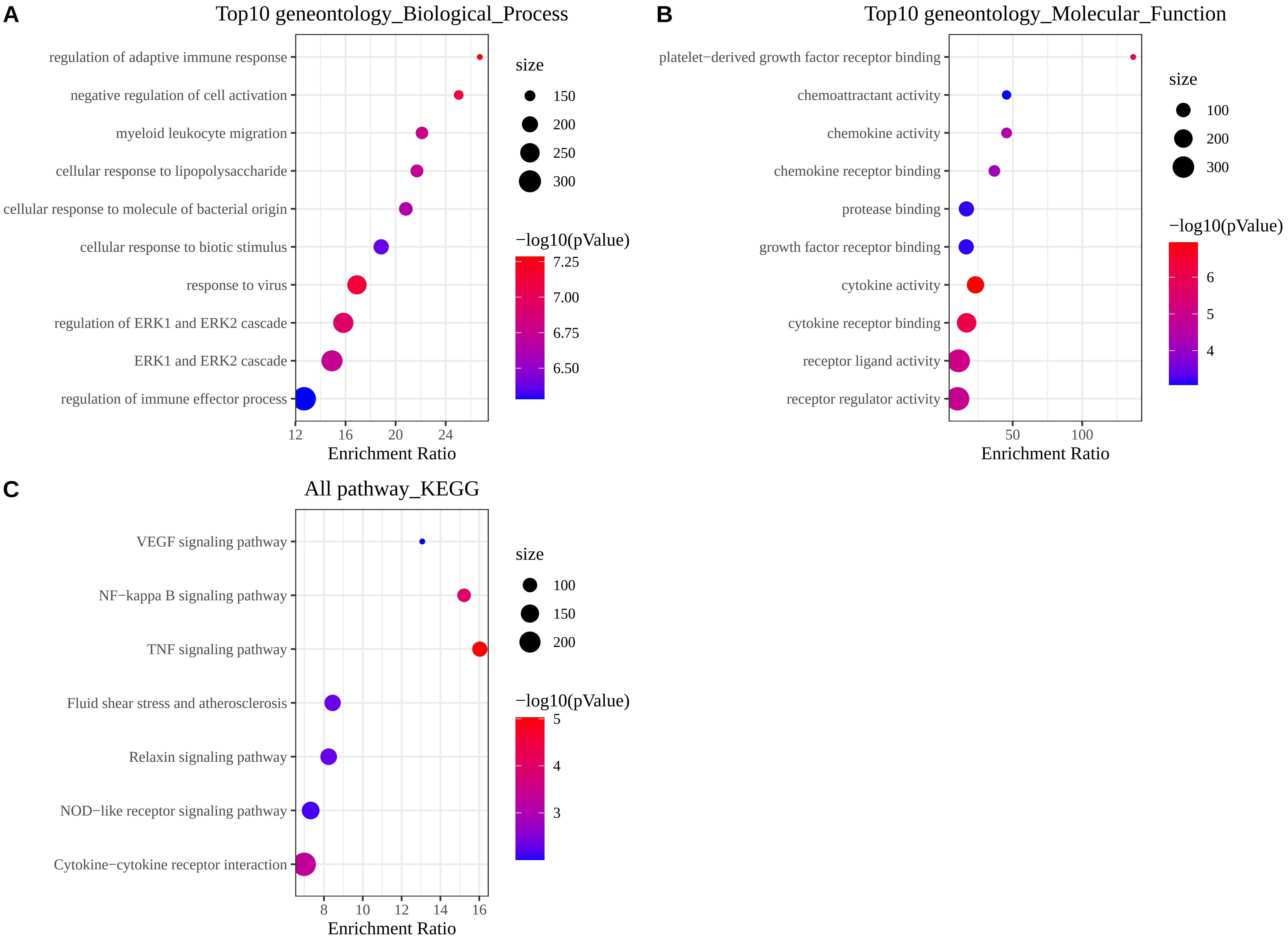
A
Top10 geneontology_Biological_Process
regulation of adaptive immune response
negative regulation of cell activation
myeloid leukocyte migration
cellular response to lipopolysaccharide
cellular response to molecule of bacterial origin
cellular response to biotic stimulus
response to virus
regulation of ERK1 and ERK2 cascade
ERK1 and ERK2 cascade
regulation of immune effector process
12 16 20 24
Enrichment Ratio
size
150
200
250
300
−log10(pValue)
7.25
7.00
6.75
6.50
B
Top10 geneontology_Molecular_Function
platelet−derived growth factor receptor binding
chemoattractant activity
chemokine activity
chemokine receptor binding
protease binding
growth factor receptor binding
cytokine activity
cytokine receptor binding
receptor ligand activity
receptor regulator activity
50 100
Enrichment Ratio
size
100
200
300
−log10(pValue)
6
5
4
C
All pathway_KEGG
VEGF signaling pathway
NF−kappa B signaling pathway
TNF signaling pathway
Fluid shear stress and atherosclerosis
Relaxin signaling pathway
NOD−like receptor signaling pathway
Cytokine−cytokine receptor interaction
8 10 12 14 16
Enrichment Ratio
size
100
150
200
−log10(pValue)
5
4
3
