# Supplementary material for: An effective prognostic model for assessing prognosis of non-small cell lung cancer with brain metastases
Source: Front Genet. 2023 Apr 13;14:1156322. doi: 10.3389/fgene.2023.1156322 (PMC10143500; doi:10.3389/fgene.2023.1156322)
Supplement: Supplementary file 1 [file DataSheet1.zip › Figure S3.pdf]

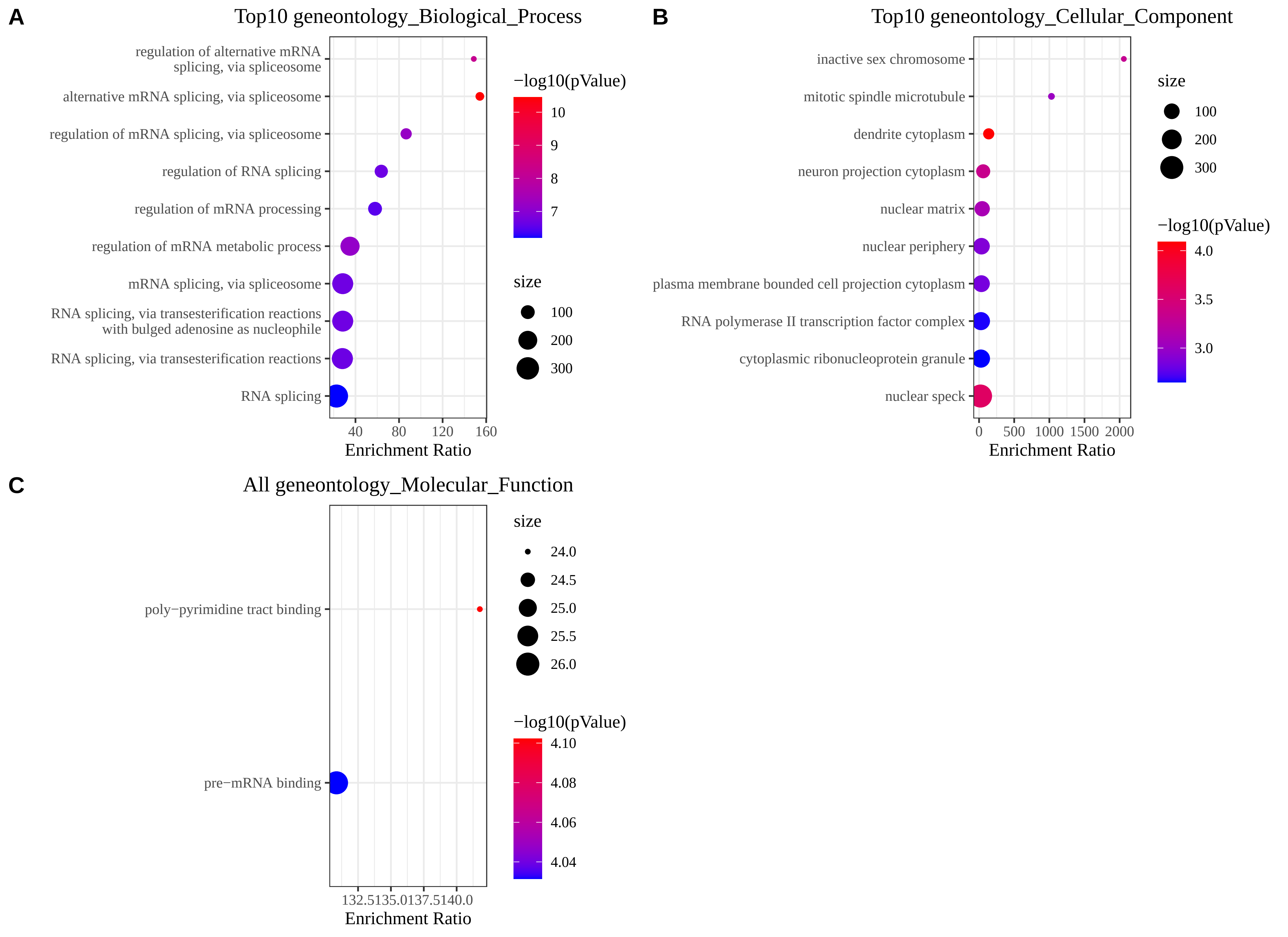
A
Top10 geneontology_Biological_Process
regulation of alternative mRNA splicing, via spliceosome
alternative mRNA splicing, via spliceosome
regulation of mRNA splicing, via spliceosome
regulation of RNA splicing
regulation of mRNA processing
regulation of mRNA metabolic process
mRNA splicing, via spliceosome
RNA splicing, via transesterification reactions with bulged adenosine as nucleophile
RNA splicing, via transesterification reactions
RNA splicing
40 80 120 160
Enrichment Ratio
−log10(pValue)
10 9 8 7
size
100 200 300
B
Top10 geneontology_Cellular_Component
inactive sex chromosome
mitotic spindle microtubule
dendrite cytoplasm
neuron projection cytoplasm
nuclear matrix
nuclear periphery
plasma membrane bounded cell projection cytoplasm
RNA polymerase II transcription factor complex
cytoplasmic ribonucleoprotein granule
nuclear speck
0 500 1000 1500 2000
Enrichment Ratio
size
100 200 300
−log10(pValue)
4.0 3.5 3.0
C
All geneontology_Molecular_Function
poly−pyrimidine tract binding
pre−mRNA binding
132.5135.0137.5140.0
Enrichment Ratio
size
24.0 24.5 25.0 25.5 26.0
−log10(pValue)
4.10 4.08 4.06 4.04
