# Supplementary material for: An effective prognostic model for assessing prognosis of non-small cell lung cancer with brain metastases
Source: Front Genet. 2023 Apr 13;14:1156322. doi: 10.3389/fgene.2023.1156322 (PMC10143500; doi:10.3389/fgene.2023.1156322)
Supplement: Supplementary file 1 [file DataSheet1.zip › Table S1.pdf]

Table S1

	High	Low	P.value		High	Low	P.value
NAV3	47	67	0.047616	FRG2B	0	8	0.007451
PCLO	37	62	0.008049	GALNT18	8	1	0.020052
CSMD2	29	51	0.011293	GPR156	8	1	0.020052
KRAS	50	33	0.044104	KIF24	1	8	0.037658
AHNAK	40	23	0.023388	MOV10	7	1	0.037025
TSHZ3	34	20	0.045947	MRPS5	7	1	0.037025
AFF2	33	18	0.028075	OR52I2	1	8	0.037658
FAM47A	16	32	0.023113	PEG10	7	1	0.037025
NLRP12	26	13	0.031577	PGLYRP3	8	1	0.020052
SLCO1C1	23	10	0.019995	PSMB11	7	1	0.037025
KCNH8	23	10	0.019995	RLF	1	8	0.037658
FN1	22	8	0.008547	SCARA5	1	8	0.037658
PCDH18	8	23	0.008784	SERPINI2	1	8	0.037658
SELP	22	11	0.049808	STK32B	7	1	0.037025
SEMA6D	25	7	0.000926	VGLL1	8	1	0.020052
SAGE1	10	22	0.044097	WDR88	1	8	0.037658
LAMB1	6	22	0.002991	ZNF81	0	7	0.01508
ANO3	21	7	0.006512	ZSWIM5	1	8	0.037658
NES	21	7	0.006512	ARHGAP4	7	0	0.007269
OR5D16	20	7	0.01033	BIRC7	7	1	0.037025
CLSTN2	7	18	0.039024	CHSY1	7	1	0.037025
CWH43	8	20	0.031511	CLEC3A	7	0	0.007269
GRM3	5	24	0.000402	DQX1	7	1	0.037025
SATB2	20	8	0.020387	FAM91A1	0	6	0.030469
ZNF835	20	7	0.01033	GLDN	7	0	0.007269
PCDH11Y	8	19	0.047032	GPRC5C	0	7	0.01508
ADAM2	7	20	0.016576	IFNAR2	0	7	0.01508
CORIN	18	6	0.012373	NAPEPLD	0	8	0.007451
IRS4	17	7	0.038337	NOP2	7	1	0.037025
RASAL2	7	18	0.039024	OR7G2	6	0	0.014765
CACNA1S	18	6	0.012373	PAPSS1	7	1	0.037025
DCAF12L[illegible]	5	18	0.00922	PAX8	7	1	0.037025
ADGRB2	17	5	0.00898	PCNXL4	7	1	0.037025
TECRL	6	16	0.048227	PHC2	7	1	0.037025
NTNG1	18	5	0.005445	PHF20	0	8	0.007451
ZNF107	6	16	0.048227	PTPRA	8	0	0.003572
C2CD3	15	6	0.047445	SLAMF7	8	0	0.003572
DOCK5	6	16	0.048227	SLC35G3	7	1	0.037025
ITGA2	15	6	0.047445	SPANXN3	7	1	0.037025
RASA1	5	16	0.024051	TGM2	7	1	0.037025
TTC14	4	16	0.010494	WISP3	7	1	0.037025
MGAT5B	5	15	0.038084	ZFP2	0	7	0.01508
CFAP58	13	4	0.027911	CYP27A1	0	7	0.01508
DYTN	4	13	0.045904	EPHX4	6	0	0.014765
PPP1R21	13	4	0.027911	FOXF2	0	7	0.01508
SHANK2	15	4	0.010232	KCNC4	7	0	0.007269

LRIT2	3	13	0.019623	PHACTR2	7	0	0.007269
MNDA	3	15	0.006704	PHF21B	0	7	0.01508
MUC6	4	13	0.045904	PROC	0	7	0.01508
ABCC2	4	13	0.045904	SLC4A7	0	7	0.01508
ARHGAP1	4	13	0.045904	SLC7A3	0	7	0.01508
ELMO1	3	13	0.019623	ST7L	0	6	0.030469
IL1RAPL2	4	13	0.045904	TMEM174	7	0	0.007269
MAP3K15	13	3	0.011259	UBC	0	7	0.01508
NXF5	14	3	0.006521	ABCD4	0	6	0.030469
OR4K17	13	4	0.027911	ARSI	0	6	0.030469
TEX14	13	3	0.011259	CECR1	6	0	0.014765
BTN2A2	3	12	0.032947	COG1	6	0	0.014765
GPR63	3	12	0.032947	CYP20A1	0	6	0.030469
HNF4A	12	4	0.045156	DGCR14	0	6	0.030469
KCNF1	3	12	0.032947	DHRS9	0	6	0.030469
NCAPG2	3	12	0.032947	DNAJB11	0	6	0.030469
TAS1R2	13	3	0.011259	EGLN3	0	6	0.030469
AMBRA1	11	3	0.032352	FUT8	0	6	0.030469
ARMC5	2	11	0.021076	GNL2	0	6	0.030469
DDI1	13	2	0.003611	GORAB	6	0	0.014765
LSAMP	2	13	0.006705	GPX6	0	6	0.030469
OR2T8	3	12	0.032947	HMP19	5	0	0.02994
RAB3GAF	3	12	0.032947	HMX3	0	6	0.030469
RASAL1	3	12	0.032947	ICE2	0	6	0.030469
RPE65	3	12	0.032947	KDM1A	0	6	0.030469
RPGRIP1L	3	12	0.032947	KERA	0	6	0.030469
THADA	11	3	0.032352	KIF3B	6	0	0.014765
WRN	3	12	0.032947	KLK7	0	6	0.030469
DOK6	2	10	0.0367	LARP1B	6	0	0.014765
PLEKHG4	2	12	0.011953	MIER3	0	6	0.030469
POLR2A	11	3	0.032352	MMP1	0	6	0.030469
SSH2	2	11	0.021076	MSLN	0	6	0.030469
ZNF516	11	3	0.032352	OSGIN1	6	0	0.014765
CYFIP2	2	10	0.0367	PDZD4	0	6	0.030469
EPHA4	11	2	0.011672	PHOSPHC	0	6	0.030469
KIF18A	10	2	0.020646	PINX1	0	6	0.030469
LZTR1	2	10	0.0367	PLA2G4F	6	0	0.014765
PKP4	2	11	0.021076	PYGO1	0	6	0.030469
SYNCRIP	12	1	0.001578	RRAGC	0	6	0.030469
UBN2	2	11	0.021076	SBSPON	0	6	0.030469
ZNF254	2	10	0.0367	SEMA4A	0	6	0.030469
ADAMTS	2	10	0.0367	SHD	6	0	0.014765
ARHGAP5	9	2	0.036067	TCN2	0	6	0.030469
CACNA1I	2	10	0.0367	TFDP3	0	6	0.030469
FBXO34	9	2	0.036067	THRAP3	0	6	0.030469
FPR2	9	2	0.036067	TNPO1	6	0	0.014765
KIAA0586	11	1	0.003013	TRBV4-1	6	0	0.014765
LTK	10	2	0.020646	USP35	0	6	0.030469

MATK	10	2	0.020646	ZDHHC6	6	0	0.014765
NXF1	9	2	0.036067	ZNF709	6	0	0.014765
OR5AC2	9	2	0.036067	AARS	5	0	0.02994
POLR3A	10	2	0.020646	ACTR3	5	0	0.02994
PRDM15	10	1	0.005714	AGXT	5	0	0.02994
SMYD3	1	11	0.005872	AURKC	5	0	0.02994
ARHGAP3	10	1	0.005714	B3GALTL	5	0	0.02994
DYRK1A	9	2	0.036067	BLID	5	0	0.02994
GRB10	8	1	0.020052	BRI3BP	5	0	0.02994
GTF3C2	9	2	0.036067	C7orf26	5	0	0.02994
GUCY2C	9	1	0.010751	CLDN4	5	0	0.02994
HTR2C	9	2	0.036067	DLG1	5	0	0.02994
MAP4K1	1	9	0.020464	FAM124B	5	0	0.02994
NDNF	9	2	0.036067	GPR162	5	0	0.02994
ZNF420	1	9	0.020464	HCLS1	5	0	0.02994
ZNF615	9	2	0.036067	HTR1D	5	0	0.02994
ZNF675	9	2	0.036067	HTRA3	5	0	0.02994
DPP3	8	1	0.020052	IFT81	5	0	0.02994
FAM110B	1	9	0.020464	IL17RE	5	0	0.02994
MALT1	10	0	0.000858	KBTBD12	5	0	0.02994
NAA15	8	1	0.020052	KDM1B	5	0	0.02994
OR11A1	1	9	0.020464	KIAA1328	5	0	0.02994
PPAPDC1	1	8	0.037658	LDLR	5	0	0.02994
PYGL	1	9	0.020464	MAGEH1	5	0	0.02994
TOP3A	1	9	0.020464	MAP1LC3	5	0	0.02994
ADGRD1	1	8	0.037658	MAP3K7	5	0	0.02994
AGBL5	1	8	0.037658	MSRB2	5	0	0.02994
ANKRD3	1	8	0.037658	NSUN6	5	0	0.02994
BFSP1	1	8	0.037658	PLA2G2F	5	0	0.02994
BTBD3	1	8	0.037658	PLS1	5	0	0.02994
CCDC27	1	8	0.037658	ST3GAL4	5	0	0.02994
FANCI	8	1	0.020052	STC2	5	0	0.02994
FBXL4	1	8	0.037658	THUMPD	5	0	0.02994
FNDC3A	1	8	0.037658	TNFRSF1	5	0	0.02994
				TRDV1	5	0	0.02994
				TRIM41	5	0	0.02994
				TRMT1	5	0	0.02994
				XXbac-BP	5	0	0.02994
				ZBTB37	5	0	0.02994
				ZCWPW2	5	0	0.02994
				ZSCAN9	5	0	0.02994
