# Supplementary material for: An effective prognostic model for assessing prognosis of non-small cell lung cancer with brain metastases
Source: Front Genet. 2023 Apr 13;14:1156322. doi: 10.3389/fgene.2023.1156322 (PMC10143500; doi:10.3389/fgene.2023.1156322)
Supplement: Supplementary file 1 [file DataSheet1.zip › Description of supplementary materials.docx]

**Supplementary materials**

**Figure S1.**

Supporting for Figure 2A. A-C, The top 10 GO terms in biological process, cellular component and molecular function level. D, The top 10 KEGG pathways.

**Figure S2.**

Supporting for Figure 2B. A and B, The top 10 GO terms in biological process and molecular function level. C, All KEGG pathways.

**Figure S3.**

Supporting for Figure 2C. A-C, The top 10 GO terms in biological process, cellular component and molecular function level.

**Figure S4.**

Supporting for Figure 2D. A and B, The top 10 GO terms in biological process and cellular component level. C, All GO terms in molecular function level. D, All KEGG pathways.

**Figure S5.**

The mutation features between RiskScore groupings. A, Mutation characteristics of different RiskScore groupings in TCGA cohort; Blue means high RiskScore group and gold means low RiskScore group. B, The difference of homologous recombination defects, fraction altered, number of segments and tumor mutation burden.

**Table S1.**

A list of 263 genes with significantly high frequency mutations between two RiskScore groups.
