# Supplementary figures and images for: An effective prognostic model for assessing prognosis of non-small cell lung cancer with brain metastases
Source: Front Genet. 2023 Apr 13;14:1156322. doi: 10.3389/fgene.2023.1156322 (PMC10143500; doi:10.3389/fgene.2023.1156322)

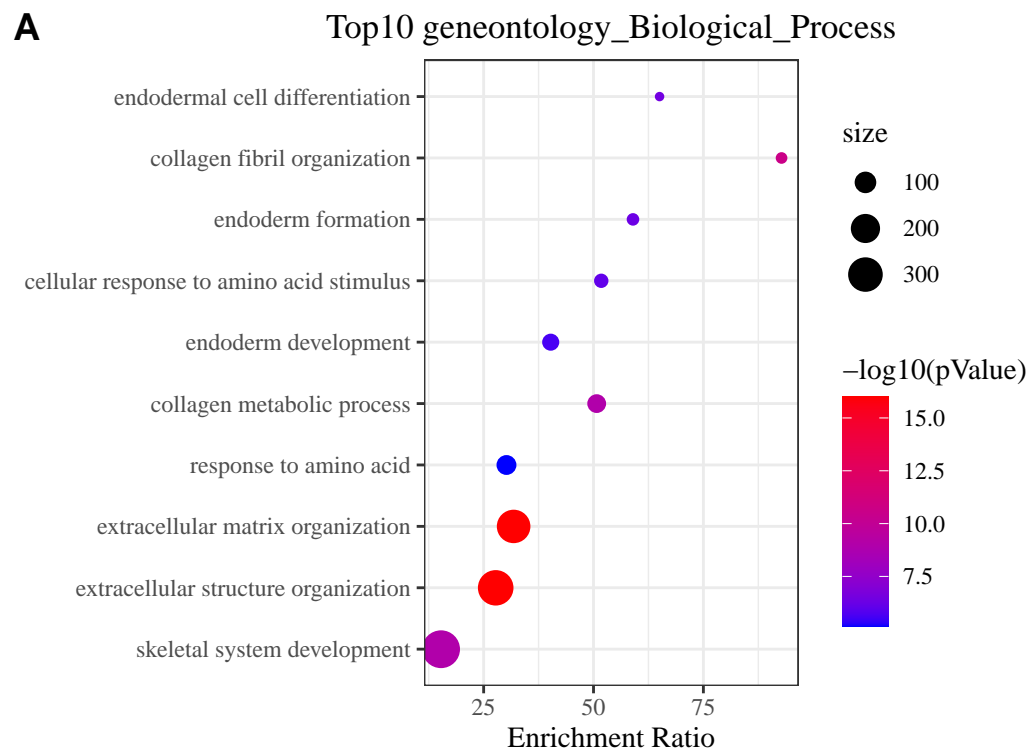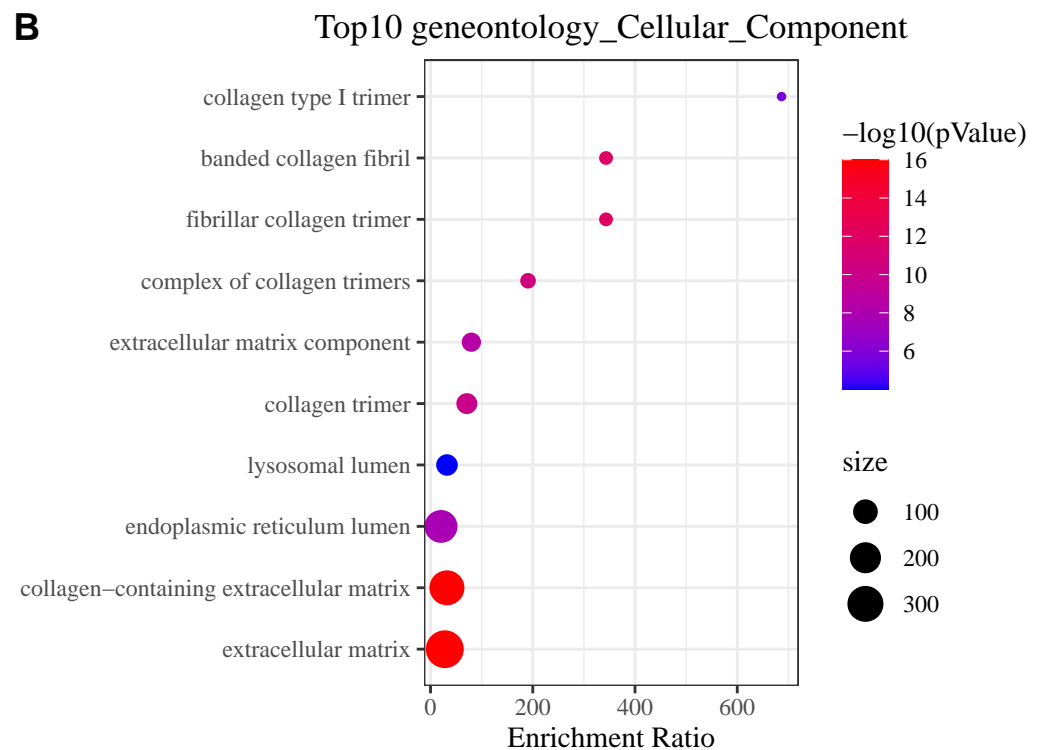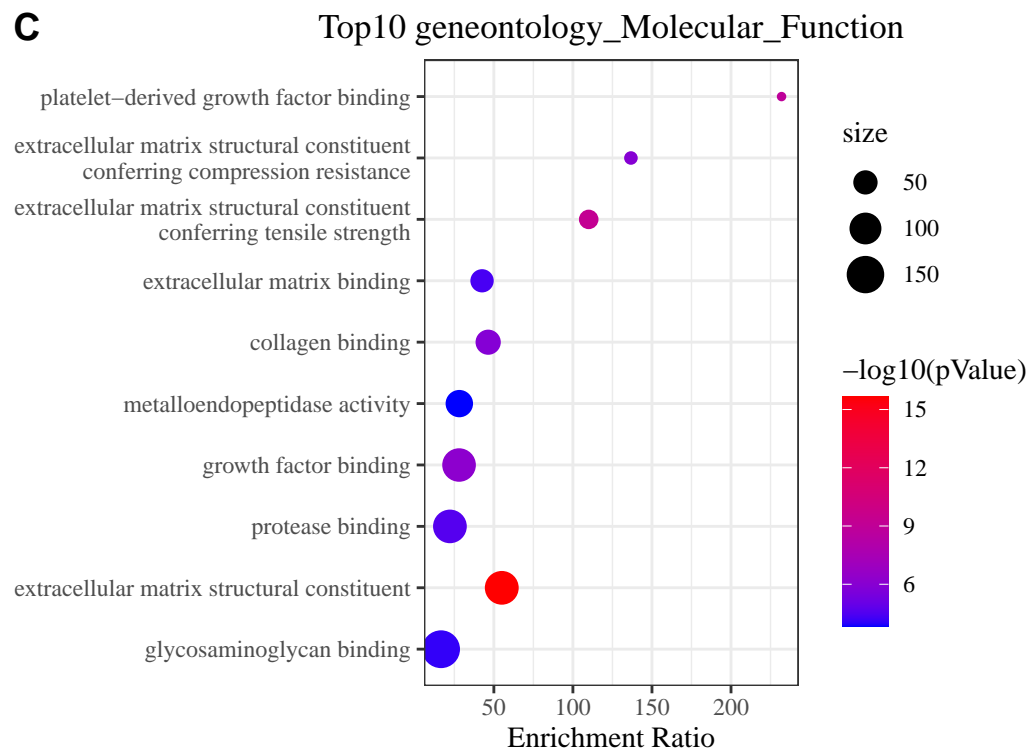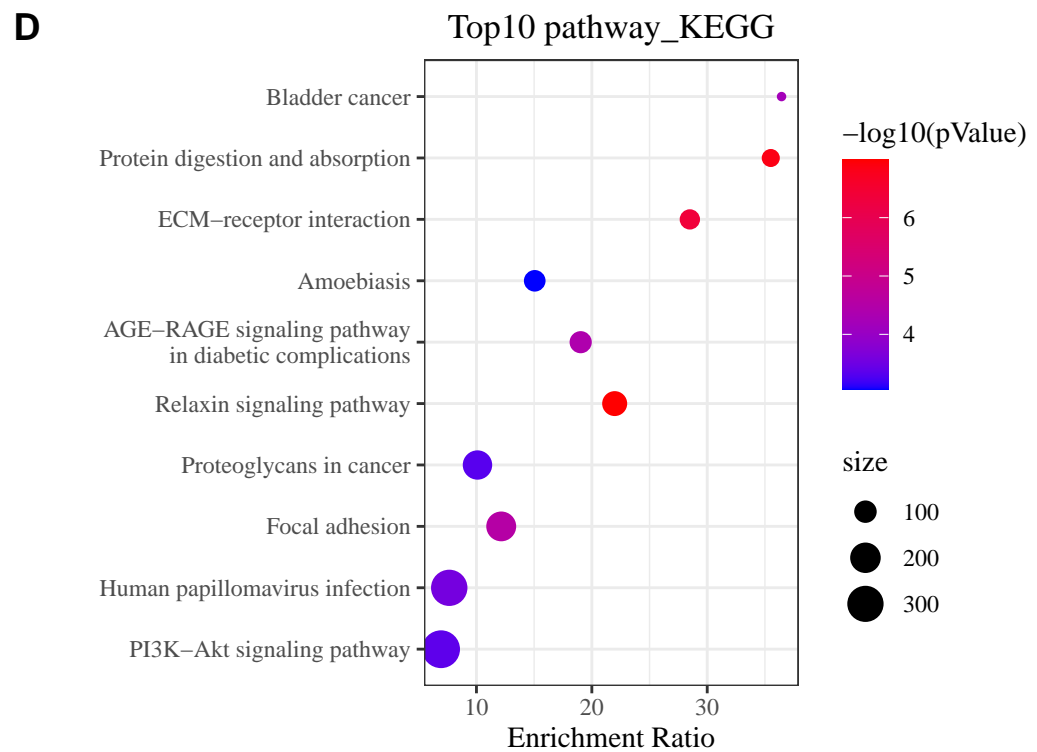

Supplement: Supplementary file 1 [file DataSheet1.zip › Figure S1.pdf]

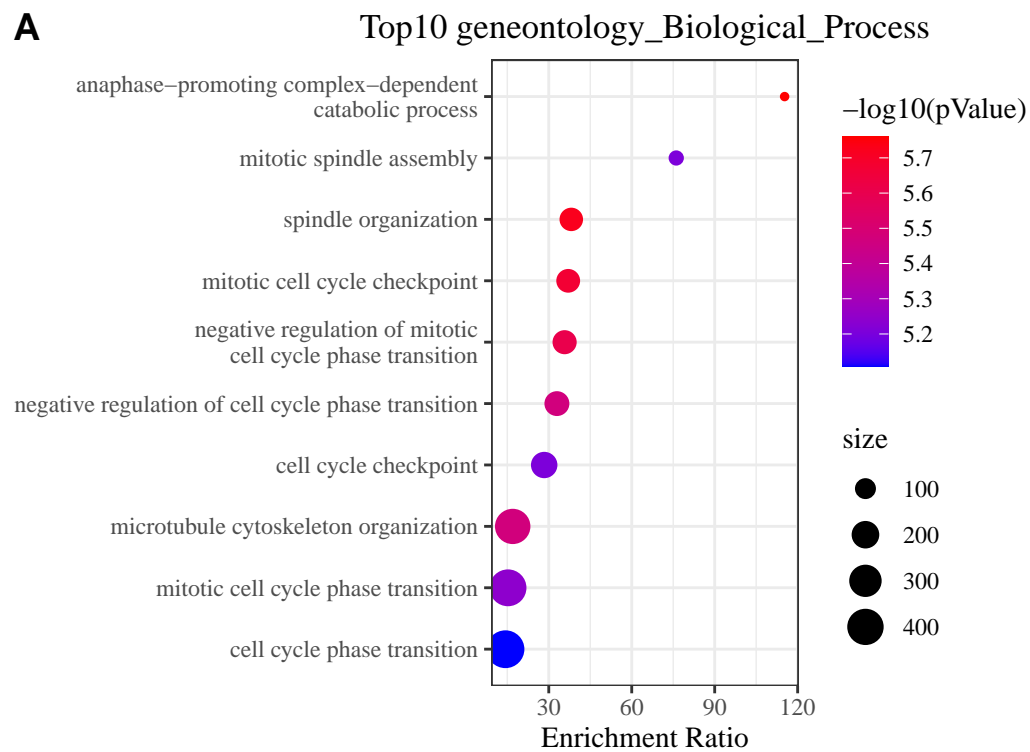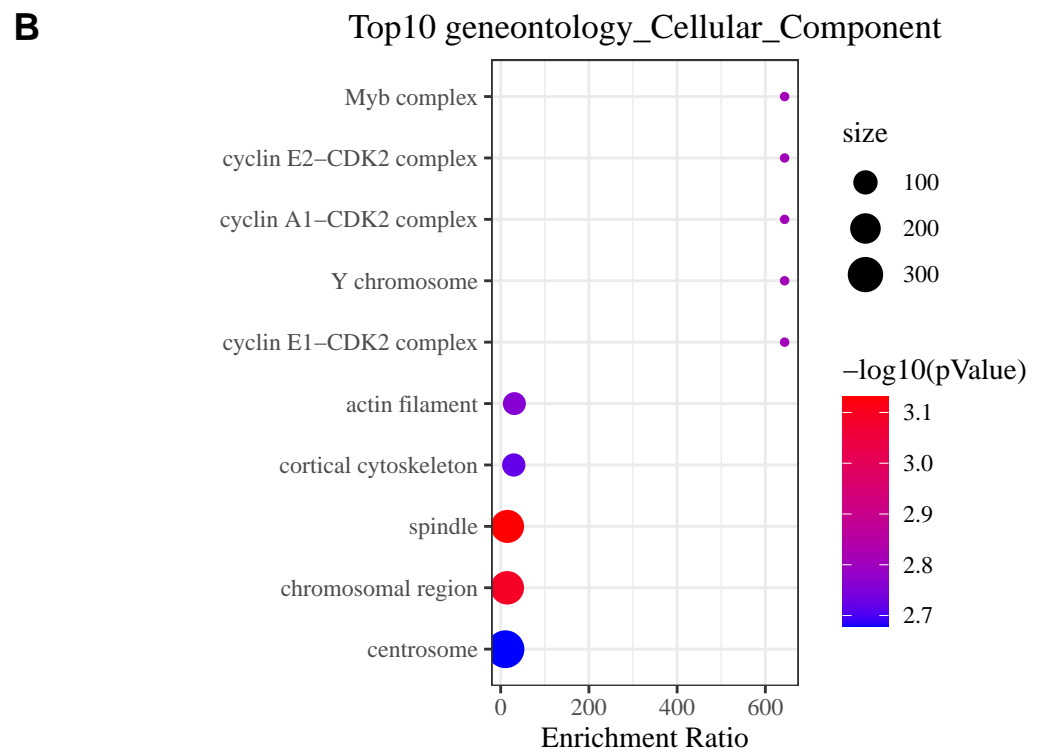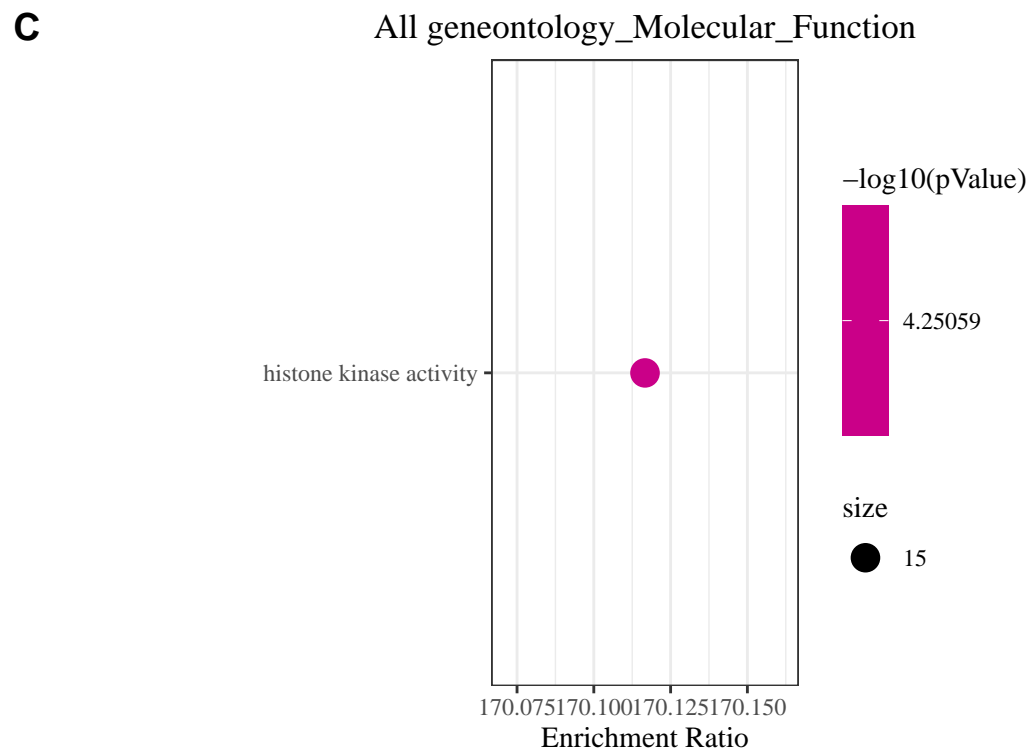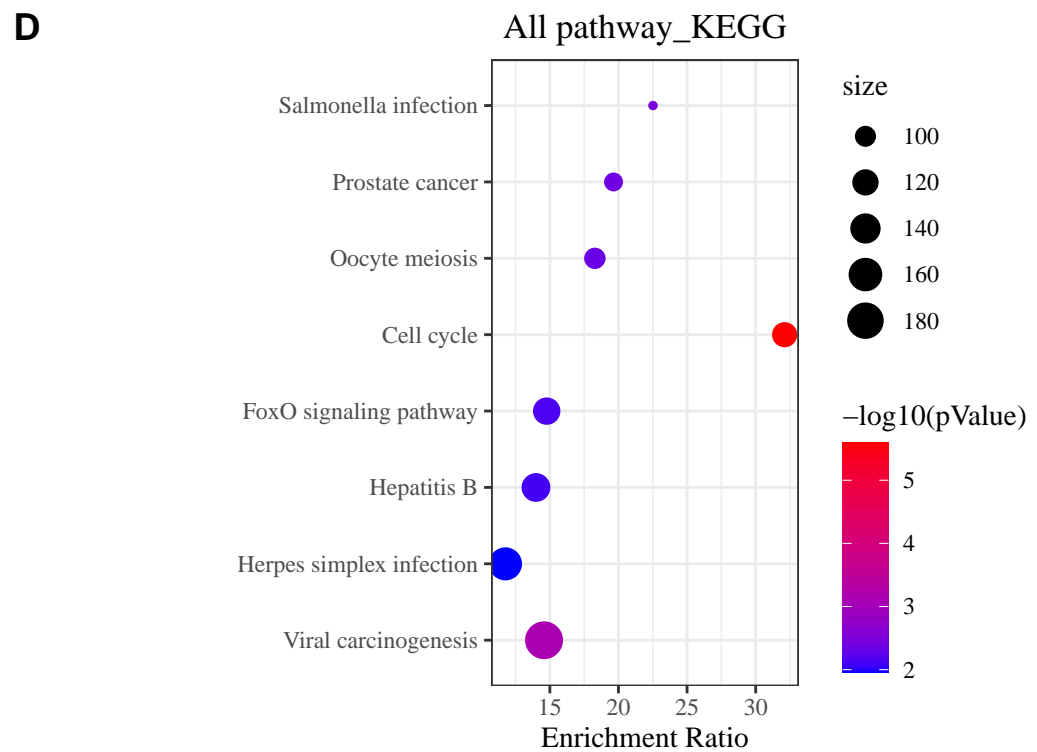

Supplement: Supplementary file 1 [file DataSheet1.zip › Figure S4.pdf]

A

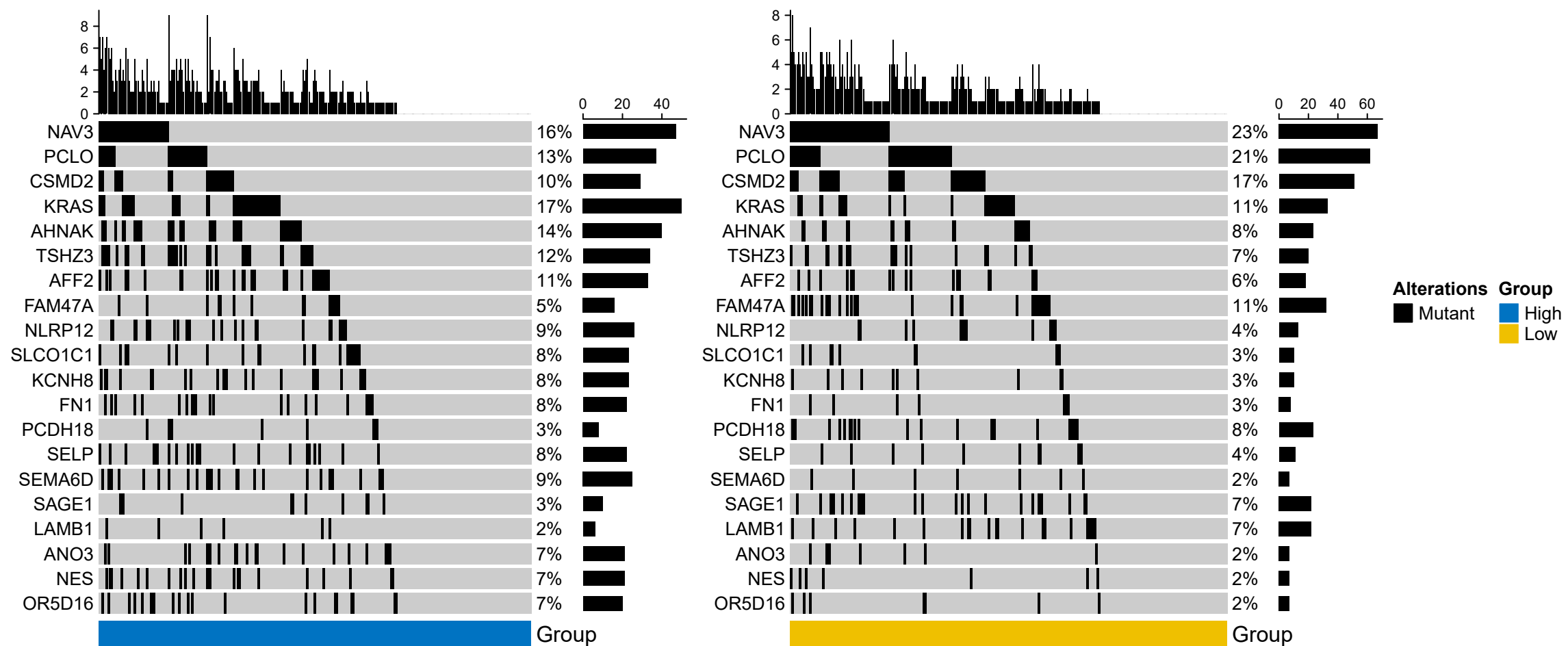

B

Group ■ High ■ Low

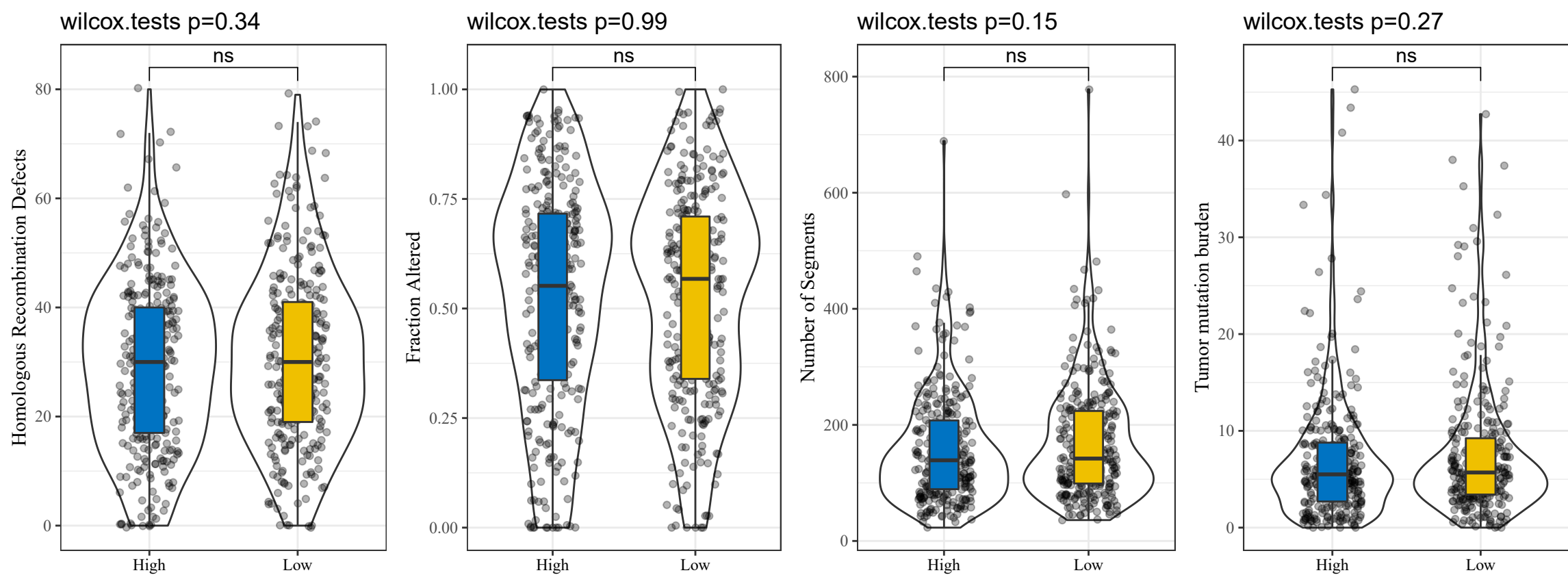

Supplement: Supplementary file 1 [file DataSheet1.zip › Figure S5.pdf]
